# A Common and Unstable Copy Number Variant Is Associated with Differences in *Glo1* Expression and Anxiety-Like Behavior

**DOI:** 10.1371/journal.pone.0004649

**Published:** 2009-03-06

**Authors:** Richard Williams, Jackie E. Lim, Bettina Harr, Claudia Wing, Ryan Walters, Margaret G. Distler, Meike Teschke, Chunlei Wu, Tim Wiltshire, Andrew I. Su, Greta Sokoloff, Lisa M. Tarantino, Justin O. Borevitz, Abraham A. Palmer

**Affiliations:** 1 Committee on Computational Neuroscience, University of Chicago, Chicago, Illinois, United States of America; 2 Department of Human Genetics, University of Chicago, Chicago, Illinois, United States of America; 3 Max-Planck-Institute for Evolutionary Biology, Department of Evolutionary Genetics, Ploen, Germany; 4 Genomics Institute of the Novartis Research Foundation, San Diego, California, United States of America; 5 Department of Pharmacotherapy and Experimental Therapeutics, School of Pharmacy, University of North Carolina, Chapel Hill, North Carolina, United States of America; 6 Department of Psychiatry, School of Medicine, University of North Carolina, Chapel Hill, North Carolina, United States of America; 7 Department of Ecology and Evolution, University of Chicago, Chicago, Illinois, United States of America; 8 Department of Psychiatry and Behavioral Neuroscience, University of Chicago, Chicago, Illinois, United States of America; Vrije Universiteit Medical Centre, Netherlands

## Abstract

Glyoxalase 1 (*Glo1*) has been implicated in anxiety-like behavior in mice and in multiple psychiatric diseases in humans. We used mouse Affymetrix exon arrays to detect copy number variants (CNV) among inbred mouse strains and thereby identified a ∼475 kb tandem duplication on chromosome 17 that includes *Glo1* (30,174,390–30,651,226 Mb; mouse genome build 36). We developed a PCR-based strategy and used it to detect this duplication in 23 of 71 inbred strains tested, and in various outbred and wild-caught mice. Presence of the duplication is associated with a cis-acting expression QTL for *Glo1* (LOD>30) in BXD recombinant inbred strains. However, evidence for an eQTL for *Glo1* was not obtained when we analyzed single SNPs or 3-SNP haplotypes in a panel of 27 inbred strains. We conclude that association analysis in the inbred strain panel failed to detect an eQTL because the duplication was present on multiple highly divergent haplotypes. Furthermore, we suggest that non-allelic homologous recombination has led to multiple reversions to the non-duplicated state among inbred strains. We show associations between multiple duplication-containing haplotypes, *Glo1* expression and anxiety-like behavior in both inbred strain panels and outbred CD-1 mice. Our findings provide a molecular basis for differential expression of *Glo1* and further implicate *Glo1* in anxiety-like behavior. More broadly, these results identify problems with commonly employed tests for association in inbred strains when CNVs are present. Finally, these data provide an example of biologically significant phenotypic variability in model organisms that can be attributed to CNVs.

## Introduction

Multiple lines of evidence suggest a potential role for *GLO1* in human psychiatric disorders. In humans, an A111E amino acid polymorphism (rs2736654) in *GLO1* has been described. The 111E allele was reported to be significantly more common in autism patients versus controls and there was also a decrease in GLO1 enzymatic activity in autistic brains [1]. A subsequent study partially replicated this finding by showing evidence that the 111A allele of *GLO1* confers protection from autism in unaffected siblings [2]. However, two more recent examinations of this and other SNPs in *GLO1* failed to replicate any association with autism spectrum disorders [3], [4]. The A111E polymorphism has also been associated with panic disorder without agoraphobia [5]. More recently, reductions in *GLO1* mRNA in the blood of mood disorder patients have been reported; patients with the same diagnosis but in remission showed normal *GLO1* mRNA levels, raising the possibility that mRNA for *GLO1* is a state-dependent marker of certain affective disorders [6]. Thus, there are multiple lines of evidence suggesting a role for *GLO1* in various psychiatric traits.

A study by Hovatta et al [7] found that high *Glo1* expression was strongly associated with high anxiety-like behavior among a panel of 6 inbred mouse strains. This correlation was bolstered by the use of viral vectors to over-express or knock-down *Glo1* expression. These studies supported a positive relationship between *Glo1* expression and anxiety-like behavior [7]. However, a different group found that mouse lines selectively bred for high anxiety-like behavior have *lower Glo1* expression and protein levels when compared to lines selected for low anxiety-like behavior [8], [9], [10]. This contradiction has raised concerns about the role of *Glo1* in anxiety-like behavior in mice [11].

Previous studies have not addressed the mechanism that gives rise to differential *Glo1* expression in mice or the location of the causal allele. We present evidence that differential *Glo1* expression in mice is the result of a common duplication of a large genomic region that includes *Glo1*. Segregating duplications and deletions in DNA are termed copy number variants (CNVs) and are a newly appreciated source of genetic diversity. It has become technically possible to identify CNVs on a genome-wide scale in humans [12], mice [13], [14], [15], [16], [17], [18], [19] *arabidopsis t.* [20], *drosophila m.* [21] and other organisms via comparative genomic hybridization. In humans, CNVs have been shown to underlie variability in expression [22] and complex traits [23]. Furthermore, while there is growing evidence that psychiatric disorders including autism [24] and schizophrenia [25] may be associated with *de novo* CNVs, the contribution of common CNVs to behavioral variability in model organisms such as mice remains poorly defined. In the present study we first identified CNVs among inbred mouse strains, and then investigated their relationship to *Glo1* expression and anxiety-like behavior.

## Materials and Methods

### CNV Analysis

We used Affymetrix Mouse Exon ST 1.0 microarrays to identify copy number differences in the genomes of inbred mice (*Mus* m*usculus*). CNV detection was accomplished through the application of Hidden Markov Modeling (HMM) [21]. We limited our analysis to regions spanning 3 probe lengths that were either duplicated or deleted in one strain relative to C57BL/6J (B6; the reference strain). We selected B6 as the reference strain because the current assembly of the mouse genome and the design of the Affymetrix Mouse Exon ST 1.0 microarray rely heavily on B6. We compared 4 strains to the reference strain: A/J, DBA/2J, LG/J, and SM/J. Two microarrays were used to represent each strain.

Genomic DNA samples from each strain were hybridized onto Affymetrix Mouse Exon ST arrays. The exon array is composed of 6,553,600 25-nucleotide-length probes with a coverage density of 4 probes for each exon of all known and predicted genes in the mouse genome. Differences in probe hybridization are used as a quantifiable measure to determine CNVs between strains. Spatial correction procedures [20] were implemented to account for confounding experimental artifacts such as local spatial bias and continuous spatial gradients. The probe hybridization intensity signal was quantile normalized [26] across all probes to further correct for systematic variance in arrays.

Each probe on the exon array was matched to its corresponding base-pair position using MegaBlast. We relied on the *Mus musculus* genome build 36.1 (Feb. 2006), obtained from the National Center for Biotechnology Information (NCBI). The annotation, and subsequent analysis, was limited to those probes for which there was a perfect alignment to a single position in the reference genome. The gene annotation was derived from NCBI's Genbank and matched to probes (natural.uchicago.edu/~tgal).

The CNV detection model used an HMM approach; predictions of deletions or duplications utilize relative hybridization intensity data for each probe. Probes representing one strain were assigned probabilities of being in one of three states: duplicated (more copies), deleted (fewer or no copies) or ground (equivalent copies), relative to a reference strain. Duplications and deletions are identified as contiguous regions of probes with high probabilities for their respective states. The unknown underlying pattern of relative deletions and duplications between strains constitutes the hidden aspect of the HMM. Parameters for the HMM were selected by training on known duplications and deletions followed by visual inspection across a range of settings to balance false positives and false negatives. The primary methods for this algorithm have been published [21]. Here we describe only the modifications of those methods used in the present study. We limited our analysis to regions spanning a minimum of 3 probe lengths that were either duplicated or deleted in one strain relative to a reference strain. Posterior probabilities for each state were calculated at all probes using a forward-backward algorithm. The forward-backward algorithm produced a biologically relevant fit to the array data and reduced the potential for over correction due to probe effects. To further reduce noise in the posterior probabilities, the algorithm's smoothing parameter was set to 0.55 for both duplicated and deleted states. The scripts for this analysis, as well as unprocessed and processed data, are all available at (borevitzlab.uchicago.edu/Members/rwilliams/copy-number-analysis/supplemental-data).

### PCR to Confirm Duplication on Chromosome 17

**Figure 1:**
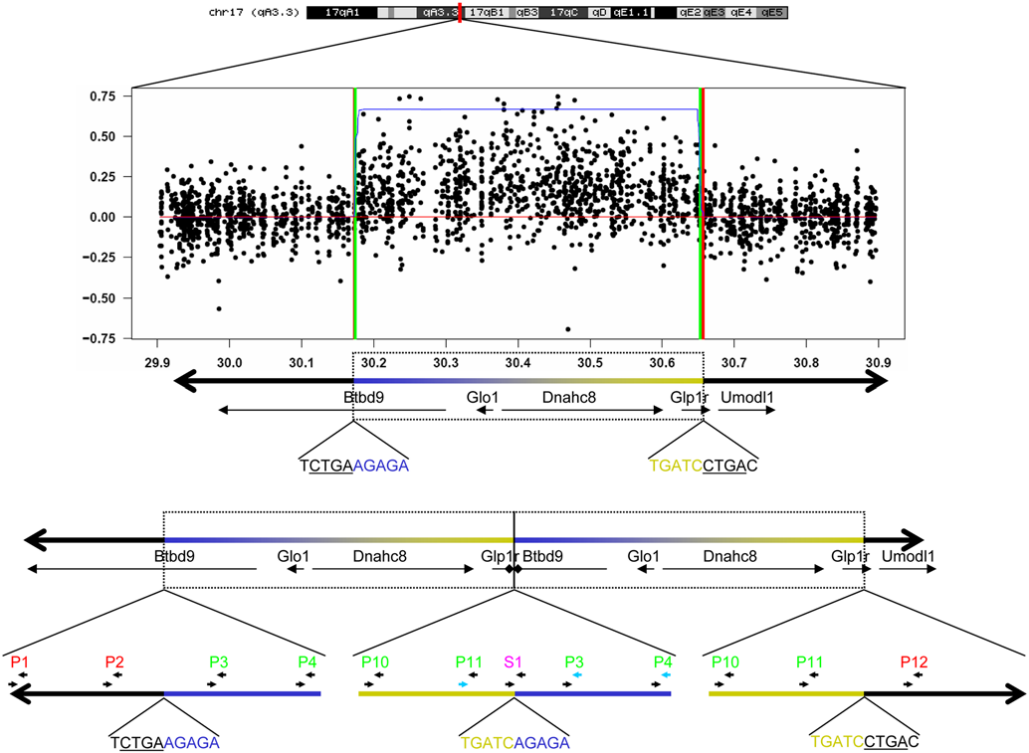
Schematic representation of the duplication. The top shows the location on chromosome 17 from 29.9 to 30.9 Mb and the duplication as identified by the HMM algorithm using hybridization intensity signals from probes (black circles) between B6 and A/J mice. The HMM predicted maximum and minimum boundaries are shown as red and green vertical lines. The duplicated region encompasses part of *Btbd9*, all of *Glo1* and *Dnahc8*, and part of *Glp1r*. The location of primers for qPCR are shown as P1, P2, etc. red or green indicates which primer pairs were outside or inside the duplication, respectively. Forward primer 11 and reverse primer 3 or 4 were successfully used to amplify a fragment across the boundary of the duplication. S1 indicates the location of primers used for sequencing. Ten bases at each critical junction are shown; CTGA (underlined) is observed on either end of the duplication.

We used the sequence from the 25-mer probes at the predicted ends of the duplications as primers for real time PCR to determine the approximate boundaries of the duplication. Primers were used to amplify regions from B6 and A/J mice using ABI SYBR® Green master mix and an ABI 7900 thermocycler in accordance with the manufacturer's instructions (Applied Biosystems, Foster City, CA). We then selected single primers from the 5′ and 3′ extremes of the putative duplication that were confirmed to be within the duplicated region by real time PCR (Figure 1); a PCR product could only be obtained from these primers if there was a tandem duplication. Based on the size of this product we then designed sequencing primers that flanked the duplication boundary. Sequencing was performed using an ABI capillary based sequencer; primer sequences are shown in Table S2.

### Ethics Statement

All research involving animal subjects was approved by the relevant institutional animal care and use committees.

### Mouse Genomic DNA

Genomic DNA from inbred mouse strains was purchased from the Jackson Laboratory (Bar Harbor, ME) or was obtained as a gift. Genomic DNA from 106 outbred CD-1 mice was obtained from animals sold by Charles River (Wilmington, MA). DNA from wild-caught mice was obtained from the worldwide collection of mouse DNAs maintained at the Max-Planck Institute for Evolutionary Biology, Germany. Our approach used only a single DNA sample from a single individual to represent each inbred strain. Both our data and those from other studies suggest the existence of copy number differences that are segregating *within* inbred strains [17]; our approach was not designed to detect segregation of a CNV within an inbred strain.

### Amygdala Gene Expression Measurements

Amygdala tissue was collected from groups of three male 10–12 week old mice from 27 different inbred strains at Genomics Institute of the Novartis Research Foundation. The amygdala was used because it has been extensively implicated in anxiety-like behavior. Mice were euthanized by cervical dislocation and decapitated. The brain was removed from the skull and positioned in a 1 mm brain block with the anterior surface abutting a single-edged razor blade placed in the first slot. The slot nearest the boundary of medulla was used as the first landmark. Double-edged razor blades were placed in slots one and two mm anterior and three mm posterior to that boundary. Two 2-mm thick sections −2 mm with respect to Bregma at posterior surface were dissected. In the first section, a horizontal cut was made at the ventral boundary of the external capsule. Another cut in line with the external capsule was made to separate the piriform cortex from the amygdala. In the remaining 2 mm thick section, the cortex was peeled apart from the hippocampus. Tissues were quickly frozen on dry ice. Tissues were pulverized while frozen, and total RNA was extracted with Trizol (Invitrogen, Carlsbad), then further processed by using the RNeasy miniprep kit according to manufacturer's protocols (Qiagen, Chatsworth, CA). The quality of all samples was verified with an Agilent Bioanalyzer (Palo Alto, CA) prior to further processing. 5 µg of total RNA was used to synthesize cDNA that was then used as a template to generate biotinylated cRNA. cRNA was fragmented and hybridized to Affymetrix MOE430 gene expression arrays using the manufacturer's protocols. The arrays were then washed and scanned, and images were analyzed and expression measurements summarized by gcRMA from the quantile-normalized probe intensities of a probe set. The 4 probe sets on the array that target *Glo1* were highly correlated (r>0.6) with each other. Individual probesets were converted to z-scores and the average of those z-scores was used to represent each strain.

### *Glo1* Expression in wild-caught mice

Gene expression was measured in whole-brain samples from six male mice from a German population (Cologne/Bonn area) and six male mice from a French population (Massif Central area) using the Affymetrix MOE430 gene expression microarrays. Each mouse was an F_1_ offspring from a male and a female that were collected directly in the wild. F_1_ offspring were used rather than individuals collected directly in the wild to avoid the effects of environmental factors (e.g. diet, age) of wild animals. In each population, the parents that gave rise to the F_1_ male originated from the same barn, or from locations in very close proximity to each other. We ensured that all of the F_1_ mice were unrelated to each other by collecting their parents from farms that were at least 1 km apart. Mating pairs were set up under standard laboratory conditions and F_1_ offspring were sacrificed at 12 weeks of age. RNA from brain was extracted and processed as described above.

### Sequencing to Obtain Additional SNPs within the Duplication

We performed *de novo* sequencing of regions within the duplication in an effort to identify additional SNPs. Because this was an exploratory effort we used only a subset of the strains examined in other parts of this study: 129X1/SvJ; C57BL/10J; C57L/J; C58/J; LG/J; NON/LtJ; NZB/BlNJ; RIIIS/J; NZW/LacJ; SJL/J; ST/bJ; BUB/BnJ; A/J; AKR/J; CBA/J; CE/J; DBA/1J; LP/J. A total of 9 previously reported SNPs were genotyped in this manner (rs33799594, rs33797803, rs33796825, rs33804194, rs33801611, rs33800133, rs33800131, rs33798567 and rs33797503). In addition, we performed sequencing across the duplication boundary and at the two ends of the duplication in an effort to obtain sequence from amplicons that would be unique in mice that had the duplication. Sequencing was performed using an ABI capillary based sequencer; selected primer sequences are shown in Table S2.

### Correlations with Behavioral Data

Behavioral phenotypes were examined using the tools at www.jax.org/phenome and were further analyzed using Statistica 5.1H (StatSoft, Tulsa, OK). Association between local SNPs and the duplication as determined using PCR was assessed using the non-parametric permutation test that is implemented at snpster.gnf.org.

### Open Field Behavior in Inbred Mice

A panel of inbred strains was tested for anxiety-like behavior in the open field; these studies were performed at Genomics Institute of the Novartis Research Foundation. A total of 901 male and female mice from 38 inbred strains were tested. The open field arena measured 43×43×33 cm (width×depth×height) and had a white Plexiglas floor and clear Plexiglas walls (ENV-515, Med Associates) that were surrounded by infrared detection beams on the X, Y and Z-axes, which tracked the animals' activity. The apparatus was isolated within a 73.5×59×59 cm testing chamber that was fitted with overhead fluorescent lighting (14 lux). All testing was conducted during the light cycle between the hours of 08:00 and 12:00. At least one hour before testing, cages were moved from the housing racks to a quiet anteroom adjacent to the testing room. Following this period of habituation, animals were removed from their home cage, immediately placed in the open field arena and allowed to freely explore the apparatus for 10 minutes. The software scored animals for a number of behaviors in the open field including total distance traveled and percent time spent in the center of the arena. These data were recorded during testing and scored in post-session analyses using commercially available software (Activity Monitor 5.1, Med Associates). Data were analyzed using a one-tailed ANOVA; a one-tailed test was justified because all previous associations (Table S4) showed that the duplication increased anxiety-like behavior. The testing apparatus was cleaned between each animal with a 0.1% bleach solution.

### Open Field Behavior in Outbred, CD-1 Mice

A cohort of 94 male, outbred CD-1 mice was tested for anxiety-like behavior in the open field; these studies were performed at The University of Chicago. CD-1 mice were obtained from Charles River (Wilmington, MA; age 4–5 weeks old) and were housed in our vivarium for 3 weeks until testing. In order to avoid false positive (type 1) errors that might occur when using outbred mice that are siblings, we specifically requested that each mouse be obtained from a separate family. The open field consisted of a clear Plexiglas container with internal dimensions of 30×30×40 cm (width×depth×height) and was lit by a small incandescent overhead light-bulb such that the center of the chamber was about 80 lux. The chamber was fully enclosed in a melamine cabinet to shield it from laboratory noise and was ventilated by a small fan on the back of the cabinet. Locomotor activity in the open filed was monitored by photo beam sensors that were arrayed in a 2 cm grid to monitor horizontal movements; the data were collected by a microcomputer and processed using software provided by the manufacturer (AccuSacn Instruments, Columbus, OH). On the test day, animals were allowed to habituate to the test room for at least 30 minutes, and were then gently placed in the center of the open field and observed for 20 minutes. We examined center time and distance traveled because the results from inbred strains suggested that these two variables would be lower in mice with the duplication. The effect of the duplication on behavior was assessed using a one-tailed ANOVA. The test chamber was cleaned between subjects with a 10% isopropanol solution.

### Measurement of Glo1 expression in Outbred, CD-1 Mice

Whole brain homogenates were prepared from 59 randomly selected CD-1 mice that had been tested in the open field study more than 2 weeks prior to tissue collection. RNA was extracted using Trizol and Qiagen RNeasy kit. Invitrogen Superscript III was used to produce cDNA according to the manufacturer's instructions. An ABI SYBR® Green master mix in conjunction with an ABI StepOne Plus machine was used according to the manufacturer's instructions to quantify *Glo1* and beta-actin cDNA expression levels. These data were expressed as fold change with respect to B6 control cDNA samples and were converted to z-scores. We observed significant test-retest variability in individual *Glo1* expression values; perhaps for this reason, we did not observe a direct correlation between behavior and *Glo1* expression. The effect of the duplication on *Glo1* expression was assessed using a one-way ANOVA.

## Results

### CNV Analysis

We identified CNVs in inbred laboratory mouse strains by hybridizing genomic DNA to GeneChip® Mouse Exon 1.0 ST Arrays, which contain probes that specifically target exons and are thus ideally suited to identify CNVs that underlie gene expression. These arrays provide at least an order of magnitude increase in probe number compared to previous efforts [13], [14], [15], [16], [17], [18]. CNVs were identified from probe hybridization intensity data using a Hidden Markov Model (HMM) approach. The HMM assigned probabilities to each probe for three states: duplicated, deleted or ground, relative to the B6 reference strain. The use of the HMM model and a large number of exon-specific probes provides high power to detect large CNVs that involve known genes; smaller or intergenic CNVs are less likely to be identified using this method. We identified a total of 68 duplications and 47 deletions (Table S1) some of which have been identified previously [13], [14], [15], [16], [17]. The limited overlap between our results and those previously published was expected because our probes did not uniformly interrogate non-genic regions. The size and frequency distribution of CNVs identified by our approach are shown in Figure S1. In this paper we focus on a duplication that is among the most consistent findings of this and previous studies of CNVs in mice [13], [14], [15], [16], [17]. This duplication is located on chromosome 17 from 30,174,390 to 30,651,226 Mb (build 36) and encompasses full copies of *Glo1* and *Dnahc8* and partial copies of *Glp1r* and *Btbd9* (Figure 1).

### PCR to Confirm Duplication on Chromosome 17

We confirmed the presence of this duplication using real time PCR to quantify genomic DNA template near the predicted ends of the duplication (Table S2) and subsequently used PCR with primers directed across the predicted duplication; such primers would only produce a product in the presence of a tandem duplication (reverse primers P3 or P4 and forward P11, see Figure 1 and Table S2). We then sequenced across the duplication boundary which allowed us to precisely define the location and the full extent of the duplication (30,174,390–30,651,226; mouse genome build 36). We used this PCR-based assay to test for the duplication in 71 inbred mouse strains, which included the 40 strains that make up the JAX phenome panel [27] (Table S3), and determined that 23 of these strains have the duplication.

### Characterization of Duplication in Wild-caught Mice

To further evaluate the history of this duplication, we examined unrelated individuals collected directly from the wild over a large geographic range from all three mouse subspecies: *M. m. domesticus*, *M. m. musculus*, and *M. m. castaneus*. PCR and sequencing at the tandem duplication boundary confirmed that *domesticus* mice from Germany (100%) and France (25%) and a single *castaneus* mouse from Taiwan (12.5%) had the duplication (Table 1). The duplication was absent in some *domesticus* populations (those from Iran), as well as most *castaneus* and all *musculus* mice and was also absent in the closely related species *M. spretus*. These results indicate that the duplication predates the isolation of laboratory mice from the wild. Whether the single observation of this duplication in a *castaneus* mouse is the result of gene flow between *domesticus* and *castaneus* populations or indicates that the duplication predates the divergence of these two populations is a important question that cannot be immediately resolved given the current data.

**Table 1:** Presence of the duplication in wild mice.

Location (*sub-species*)	Number with duplication/total number observed
Germany (*dom*)	18/18
France (*dom*)	4/16
Iran (*dom*)	0/8
India (*cast*)	0/8
Taiwan (*cast*)	1/8
Czech Republic (*mus*)	0/8
Kazakhstan (*mus*)	0/8
*Mus spretus*	0/18

Table showing the location of wild mouse collection and the fraction of total mice observed that tested positive for the duplication. The assay used does not distinguish between mice that are heterozygous for the duplication and those that are homozygous; both conditions are scored as positive. Abbreviations: “*dom*”: *Mus musculus domesticus*; “*cast*”: *Mus musculus castaneous*; “*mus*”: *Mus musculus musculus*. *Mus spretus* is a different species that is closely related to *Mus Musculus*.

### Relationship between Duplication and Gene Expression in BXD RI Strains

**Figure 2:**
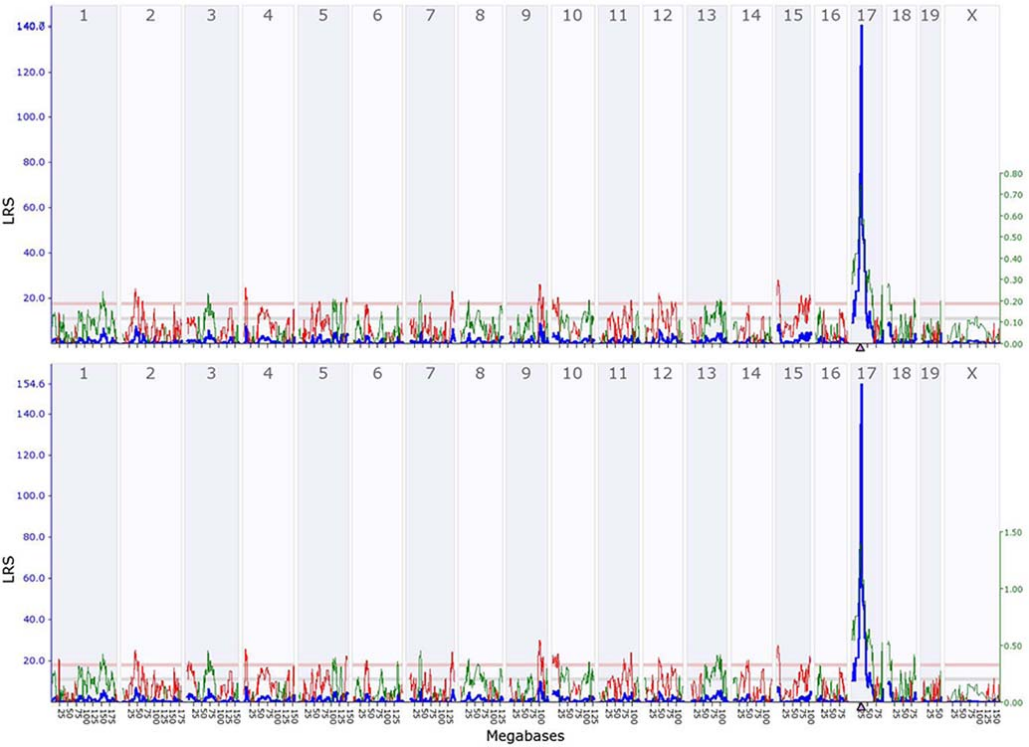
cis-eQTL for *Glo1* and the probe 1458719_at. Plot shows mouse genome on x-axis and log ratio score (LRS) on y-axis. Horizontal lines indicate genome-wide suggestive and significance thresholds as determined by permutation. *Glo1* (1424109_a_at) (panel A) and the probe 1458719_at (panel B) measured by MOE430v2 microarray using hippocampal tissue as described elsewhere (generated by www.genenetwork.org).

We next explored the possibility that this duplication causes heritable gene expression differences. We used WebQTL (www.webqtl.org) to explore the relationship between the duplication and *Glo1* expression in the BXD recombinant inbred (RI) lines. The BXD RI lines are a cross of DBA/2J, which has the duplication and B6 which does not have the duplication. We identified highly significant cis-eQTLs for all 4 probesets that target *Glo1* (Figure 2A), as well as probe set 1458719_at which has been annotated as either *Btbd9* or *Glp1r* (Figure 2B) in hippocampal expression data (Hippocampus consortium M430v2 (Jun06) PDNN). We observed similar eQTLs in all other tissues for which expression data in the BXD RI strains was available (whole brain, striatum, cerebellum, eye, hematopoietic cells, kidney and liver).

The probeset 1458719_at maps to an intron of *Glp1r* and is homologous to several mouse ESTs (BM233846, BQ560923, BX632944). These ESTs appear to be fusion products of the duplicated copy of *Btbd9* and an intron of *Glp1r*; mRNA transcription presumably continues across the duplication boundary and incorporates sequence from an intronic region of *Glp1r*, thus producing a novel gene product in strains that possess this duplication. Consistent with this hypothesis, we have only observed appreciable signal from 1458719_at in strains that are positive for the duplication. We did not observe any significant cis-eQTLs in any of the tissues that we examined for the many probes that correctly measure the partially duplicated genes Btbd9 and Glp1r nor did we observe evidence of significant eQTL for Dnahc8. Thus, while 2 genes are fully duplicated (Glo1 and Dnahc8) and two others are partially duplicated (Btbd9 and Glp1r), only Glo1 shows a statistically significant increase in expression as a result of the duplication.

### Relationship between Duplication and Gene Expression in a Panel of Inbred Strains

Having established that an eQTL for *Glo1* was highly significant when considering a cross between two inbred strains, we sought to extend these findings by mapping an eQTL for expression of *Glo1* in a panel of inbred strains, similar to approaches that have been proposed for genome-wide association analysis [28] and in an effort to follow up on the findings of Hovatta et al [7]. We obtained expression data from the amygdala for 27 inbred strains for which duplication status was also known. To our surprise, of the 48 SNPs examined between 30 and 31 Mb neither single SNPs nor 3-SNP haplotypes were strongly associated with *Glo1* expression in the amygdala. Specifically, the maximum −log_10_(p) for any single SNP was 2.9 (rs3150712; 30.49 Mb) whereas the maximum −log_10_(p) for any 3-SNP haplotype was 2.77 (rs33190587; 30.12 Mb). When the same tests were re-run with a correction for population structure, the highest scoring SNP and 3-SNP haplotypes did not change, but their −log_10_(p) decreased to 2.15 and 2.28, respectively. In contrast, the presence of the duplication, as determined using our PCR-based assay, was extremely significantly associated with *Glo1* expression (−log_10_(p)>6), revealing the expected cis-eQTL for *Glo1*. Correction for population structure did not diminish this extremely significant result. These data were surprising because we had expected that SNPs and 3-SNP haplotypes in the vicinity of the duplication would be strongly associated with the duplication and hence expression of *Glo1*.

### Haplotype Structure Among Inbred Strains from 30 to 31 Mb

**Figure 3:**
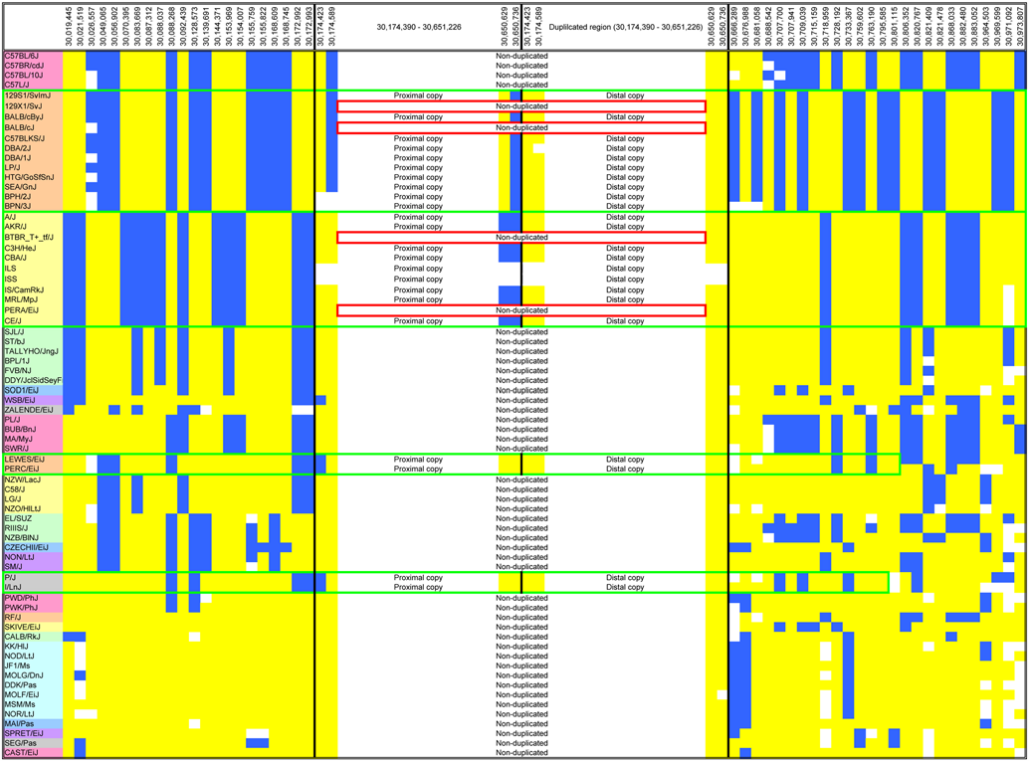
Haplotype blocks from 30–31 Mb across 71 inbred strains at 48 SNP markers. The duplicated region is denoted by heavy black vertical lines; duplicated strains are denoted with two repeating regions labeled ‘proximal copy’ and ‘distal copy’, whereas non-duplicated strains are indicated with a single box that contains the text ‘non-duplicated’. The major and minor alleles are coded as yellow (major) and blue (minor), respectively. Missing data at a given SNP is indicated in white. Strain names are shaded to indicate haplotype identity. Green boxes indicate haplotypes that contain the duplication. Red boxes (129X1/SvJ, BALB/cJ, BTBR_t+_tf/J, PERA/EiJ) indicate strains that belong to duplication containing haplotypes but do not have the duplication; we suggest that these strains have undergone reversions to the non-duplicated state. Within the duplicated region it was possible to genotype 4 SNPs (30,174,423; 30,174,589; 30,650,629; 30,650,736) that have been duplicated and to uniquely determine their genotype at both locations, as described in the text, these SNPs occur twice in the duplicated strains. Marker position (Build 36.1) and strain ID are indicated.

SNP-association results from the BXD RI strains, but not from a panel of inbred strains, identified a cis-eQTL at the location of the duplication for *Glo1*. To better understand this surprising result, we examined the haplotype structure from 30–31 Mb in the 71 strains where the duplication status was known using the same 48 SNPs. Significantly, these SNPs flanked, but were not internal to the duplicated region. We observed four distinct haplotypes that contained the duplication (Figure 3, denoted with green borders). Careful examination of the data in Figure 3 led us to conclude that multiple non-allelic homologous recombination events had taken place *within* the duplicated region. Non-allelic homologous recombination can occur between two chromosomes that both contain the duplication or between one duplicated and one non-duplicated chromosome. Such a recombination would lead to the exchange of either the distal or proximal haplotypes shown in Figure 3 without altering duplication status. In particular, this explanation accounts for the SNP haplotype observed in the proximal regions of B6 and related strains (not duplicated) and DBA/2J (duplicated; Figure 3). Thus, we hypothesize that recombination within the duplication is at least partially responsible for the complex haplotype structure around the duplication.

### Sequencing to Obtain Additional SNPs within the Duplication

The number of SNPs located within the duplication that were available from public databases was relatively small compared to the number of SNPs on either side of the duplicated region. Those that were available had higher than average rates of missing genotypes, and genotypes appeared to be disproportionately missing in strains that were positive for the duplication. We suspected that both phenomena were due the SNP assays or strain-specific SNP genotypes being scored as failures because heterozygous genotypes were obtained from inbred strains. A heterozygous genotype contradicts the assumption that these mice are inbred, and could thus appear to be a technical error. We suspected that these apparently heterozygous genotypes weredue to polymorphisms between the proximal and distal duplicated regions. To test this hypothesis, we sequenced 9 SNPs that were entirely within the duplication in 18 inbred strains (6 of which had the duplication). We observed multiple apparently heterozygous genotypes in duplicated strains, but none in non-duplicated strains (data not shown). The heterozygous genotypes can best be explained by a region that is polymorphic when comparing the proximal and distal copies of the duplication, rather than being heterozygous due to differences between two chromosomes. The heterozygous genotypes agreed with the haplotype structure shown in Figure 3, and it was possible to identify the haplotypes of the proximal and distal copies. These observations are consistent with the hypothesis that the complex relationship between the duplication and surrounding haplotypes is due to crossovers that occurred within the duplicated region.

In order to avoid the ambiguity associated with heterozygous genotypes we used sequencing primers that spanned the duplication boundary (duplicated strains only). For comparison, we also used sequencing primers where one primer was outside of the duplicated region and the other was inside the duplicated region (all strains). This allowed us to uniquely compare SNPs that might be polymorphic between the proximal and distal copies in the duplicated strains, and also to directly extend our haplotype analysis into the duplicated region. When considering the proximal duplication boundary (∼30.17 Mb), we observed two informative SNPs (30,174,423 and 30,174,589) both of which appear to have been exchanged via crossing over between duplicated and non-duplicated strains. The region near to 30.65 Mb was more interesting: a SNP at 30,650,629 is polymorphic such that the second duplication-containing haplotype block (the one that includes the A/J strain) differs from all other duplicated strains. The second duplication-containing haplotype block has a characteristic allele that is never observed in the analogous region in any of the other duplicated or non-duplicated strains. The second SNP in this region (30,650,736) perfectly distinguishes the internal from the external duplicated and non-duplication sequence and is thus never observed in non-duplicated strains. Both observations are consistent with these SNPs arising after the duplication occurred, though we cannot exclude the possibility of recombination or gene conversion with an unobserved, non-duplicated chromosome that contained these SNPs. Had the SNP at 30,650,736 been present in the inbred strain database, it would have performed as well as our PCR assay in terms of predicting the presence of the duplication; thus, exhaustive SNP coverage may help to address problems with inbred strain haplotype mapping, but will also increase the multiple testing burden and thus are unlikely to offer a solution to the problem of using SNPs to identify CNV among inbred strain panels.

### Evidence for Recent Loss of the Duplication

Another observation that we made in examining the data in Figure 3 is that four strains (129X1/SvJ, BALB/cJ, BTBR T+tf/J and PERA/EiJ) appeared to belong to one of the two common duplication-containing haplotypes but did not contain the duplication (Figure 3; denoted with red boxes). We hypothesized that the duplication has reverted to the non-duplicated state in these strains by a process of non-allelic homologous recombination (sometimes termed unequal crossing over). This hypothesis was confirmed both by examination of DNA samples obtained from previous generations of these inbred strains as well as by taking advantage of the known breeding history of these strains. In this regard, one compelling case was the absence of the duplication in BALB/cJ. Historically, we know that BALB/cByJ and BALB/cJ were separated in the 1930s [29] but they remain isogenic at all typed SNPs and clearly share the same haplotype throughout the duplicated region (Figure 3). The absence of the duplication in BALB/cJ could be explained by a reversion event that occurred after their separation from BALB/cByJ in 1937. We obtained DNA samples from JAX for BALB/cByJ from 1982 and 2000 and determined that both had the duplication. We also obtained DNA from BALB/cBy (used by Tafti et al [30], see below), which was separated from BALB/cByJ in the 1970s, and found that, unlike BALB/cByJ, BALB/cBy did not have the duplication. Finally, we obtained DNA from 1998 and 2006 for BALB/cJ and found that the duplication was not present in either sample. These data are consistent with the hypothesis that the duplication has reverted to the non-duplicated state and/or that duplicated and non-duplicated alleles have been segregating within the BALB/c-lineage for some time. Because of the known breeding history of the BALB/c mice, the reversion observed in 129X1/SvJ, which shares the BALB/c haplotype, is presumed to have an independent origin. We obtained a sample from the F_30_ generation of PERA/EiJ (1982) and found that this sample was positive for the duplication, whereas the modern samples (F99) were negative for the duplication. SNPs that were inside the duplication in the F_30_ sample of PERA/EiJ exactly matched the other strains in that haplotype block (data not shown). These observations are consistent with loss of the duplication due to unequal crossing-over, as discussed above. Genomic DNA from BTBR T+tf/J from 1996 and 2004 as well as a sample from 129X1/SvJ from the 1990s were similar to modern samples and thus offered no further insights.

Thus, this duplication cannot be reliably predicted based on single SNPs or multi-SNP haplotypes due to simple recombination, genotyping problems due to heterozygous genotypes, and reversion to the non-duplicated state via non-allelic homologous recombination. The observation of changes in CNVs over time are consistent with recent reports from Watkins-Chow & Pavan [18] and Egan et al [17] which show changes in CNVs among and within inbred strains. Indeed, Egan et al [17] appears to have identified a copy number difference for the duplication discussed in this paper between two different A/J mice (denoted as INTRA-1 in Supplemental Table 1c on page 76 of the supplemental materials for that paper).

### Relationship between the Duplication and *Glo1* Expression in Wild-Caught Mice

We examined gene expression in whole brain homogenates from unrelated, outbred, wild-caught *domesticus* mice from Germany (6 mice) and France (6 mice). We found a highly significant (−log_10_(p)>4) association between the presence of the duplication, as determined using our PCR-based assay, and expression of *Glo1* as measured by all four *Glo1*-specific probesets as well as 1458719_at.

We examined expression levels of all 4 *Glo1* probesets as well as 1458719_at in a previously published dataset [31] that examined inbred wild-derived *domesticus*, *musculus* and *castaneus* strains. Even with a very small sample size (n = 2 per group) *Glo1* expression was significantly higher in the *domesticus*-derived inbred strains (−log_10_(p)>2.25) relative to the other two inbred strains. PCR-based genotyping of genomic DNA confirmed that only the *domesticus*-derived inbred strain had the duplication. These data further support a direct relationship between the duplication and *Glo1* expression in outbred, wild-caught and wild-derived inbred mouse strains.

### Relationship between the Duplication and Anxiety-Like Behavior Using Publicly Available Datasets

Our data clearly establish that increases in *Glo1* expression in panels of inbred strains, outbred populations and even in wild-derived populations are driven by a duplication of the *Glo1* gene. This knowledge should facilitate an examination of this gene's role in behavioral phenotypes. We used the results from our PCR-based genotyping method to score presence or absence of the duplication in many common inbred strains using data shown in Table S3 and found significant correlations with various classical tests of anxiety-like behavior including the elevated zero maze, elevate plus maze, light dark box and open field test that were available from the JAX Phenome site (www.jax.org/phenome). A partial summary of these findings is presented in Table S4. For example, data from 8 strains tested in the elevated zero maze [32] showed that the duplication was associated with fewer beam breaks in the closed quadrant of the open field and more fecal boli (r = 0.82; p = 0.01, r = 0.78; p = 0.023, respectively). Data from another set of 13 strains tested in elevated zero maze study (Brown1; unpublished) showed that the duplication was associated with greater numbers of fecal boli (r = 0.67; p = 0.034). Data from 13 strains tested in the elevated plus maze (Brown1) again showed that the duplication was associated with a greater number of fecal boli (r = 0.73; p<0.01). Data from 13 strains tested in the light dark box (Brown1) showed that the duplication was associated with less total activity and a greater number of fecal boli (r = 0.76; p<0.01, r = 0.83; p<0.01, respectively). Data from 21 strains tested in the open field test [33], [34] showed that the duplication was associated with less movement in the open field for each of the first 5 minutes (e.g. distance traveled in the first minute: r = 0.57; p<0.01). Data from a separate study of 8 strains also tested in the open field [33], showed that the duplication was associated with less activity and time in the center of the open field (r = 0.89; p<0.01 and r = 0.84; p = 0.010, respectively). Data from a third study of the open field (Brown1; unpublished) showed that the duplication was associated with less activity and a greater number of fecal boli (r = 0.73; p<0.01, r = 0.67, p<0.013, respectively). It is important to note that in all cases, presence of the duplication was negatively associated with activity and positively associated with anxiety-like behavior, consistent with our hypothesis and the observations of Hovatta et al [7]. A number of other phenotypes were also correlated with the presence of the duplication even though they have no obvious relationship to anxiety. For example, latency to respond to the hot plate test, a measure of nociception [35], was slower in strains that carried the duplication (r = 0.81; p<0.01). Taken together these data demonstrate that the duplication is correlated with behaviors including, but not limited to, those associated with anxiety-like behaviors.

### Relationship between the Duplication and Anxiety-Like Behavior Using New Inbred Strain Data

**Figure 4:**
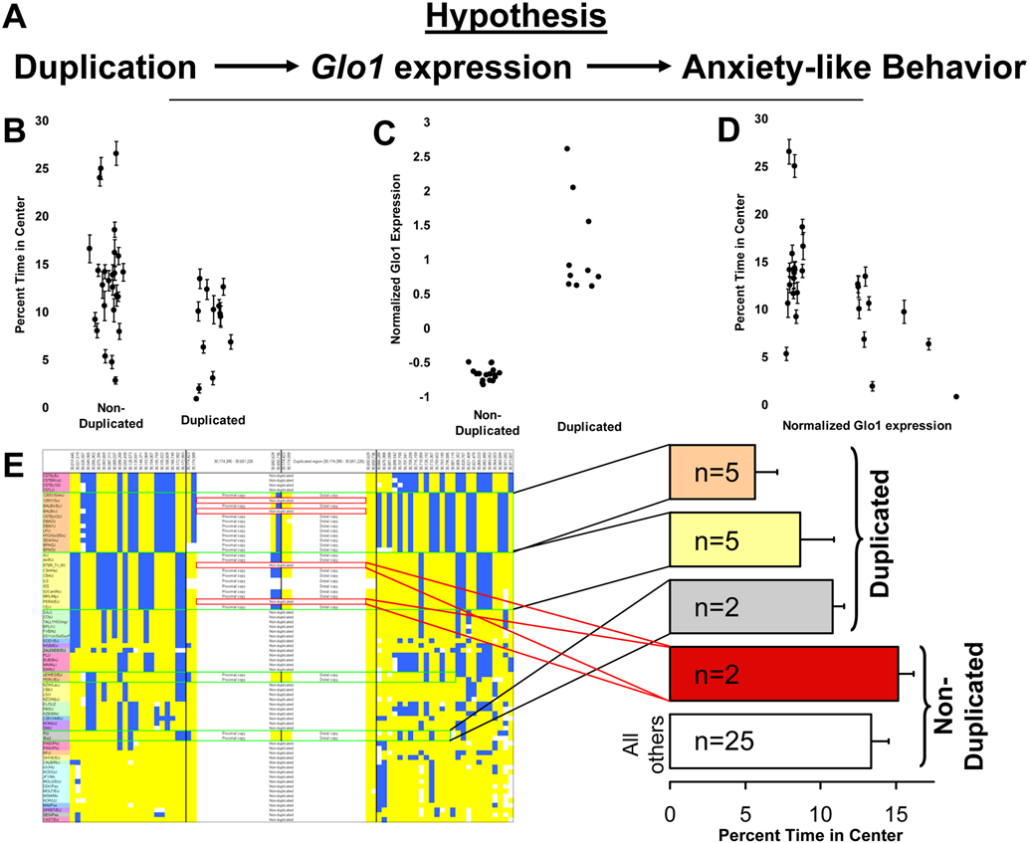
Relationship between behavior, duplication, gene expression and haplotype block. A) hypothesized relationship between the duplication, *Glo1* expression and anxiety like behavior; B) Correlation between duplication status and anxiety-like behavior as measured by percent time in the open field and presence of the duplication. A total of 901 male and female mice from 38 inbred strains were tested (n = 5–42 per strain). Random scatter along the x-axis and within duplicated and non-duplicated groups has been added. C) Correlation between duplication status and normalized gene expression in the amygdala from 27 inbred strains. Random scatter has again been added along the x-axis. D) Correlation between normalized *Glo1* expression in the amygdala from 27 inbred strains and anxiety-like behavior as measured by percent time in the open field. E) Relationship between haplotype blocks and anxiety-like behavior as measured by percent time in the open field. Behavioral data were obtained from multiple individuals from each of the indicated number of strains, error bars represent the standard error for the mean of strains, not individuals, which would have been much smaller, but statistically inappropriate because the unit of analysis is strains not individuals.

Because of the limited number of inbred stains for which behavioral data were available in the public databases (21 or less strains per study), and the genetic and environmental variability presumed to exist for anxiety-like behavior, the power of the correlations observed in public databases was limited. To more rigorously test the association between the duplication and anxiety-like behavior, we used our own behavioral data from 38 inbred strains (901 individual mice total) for which the duplication status was known. We observed a significant reduction in percent time in the center of the open field among strains that had the duplication (p = 0.0043), further demonstrating that the duplication was associated with greater anxiety-like behavior (Figure 4B). The strength of this association was not changed when a correction for population structure available in SNPster (snpster.gnf.org) was applied, suggesting that this association was not due to artifacts associated with population structure. While none of these correlations would have been significant if a correction for multiple comparisons (e.g. all CNV identified in this study) had been applied, such corrections were unwarranted because we had a strong prior hypothesis that *Glo1* expression affects anxiety-like behavior. In the present study we have focused on a single CNV, and so the required threshold has been set to the traditional value of 0.05.

### Relationship between *Glo1* Expression and Anxiety-Like Behavior in Inbred Strains

Because our hypothesis is that the duplication increases gene expression and thus alters behavior, we also examined the relationship between the duplication and *Glo1* expression in the amygdala (Figure 4C; p<0.000001) and the relationship between *Glo1* expression in the amygdala and anxiety-like behavior (Figure 4D; p = 0.0012). Both correlations were more significant than the correlation between the duplication and behavior. We used multiple regression to examine the relationship between the duplication, Glo1 expression and behavior. Both forward- and reverse-selection methods arrived at a model that included expression but did not include our PCR-based measure of the duplication. This might be attributed to some strains having more than two copies of the duplicated region and showing correspondingly higher expression; our PCR-based technique does not determine the number of extra copies of this region. These results are consistent with the hypothesis that expression of *Glo1* is a better predictor of behavior than the duplication itself.

### Relationship between Flanking Haplotypes and Behavior in Inbred Strains

An alternative hypothesis that might also explain the correlation between the duplication and anxiety-like behavior is that the functionally significant allele is genetically linked to, but not contained within, the duplication. To test this hypothesis, we calculated the average anxiety-like behavior associated with three of the four duplication-containing haplotypes identified in Figure 3 for which our study of 38 inbred strains provided corresponding behavioral data (Figure 4E). We found that each of the three duplication-containing haplotypes was associated with higher anxiety-like behavior compared to the average anxiety-like behavior associated with all non-duplicated strains, which is consistent with the hypothesis that the duplication alters behavior, but is inconsistent with the alternative hypothesis that the duplication is genetically linked to another allele that alters anxiety-like behavior. We considered separately the two strains that were members of the duplication containing haplotype blocks but had apparently lost the duplication (BTBR_T+_tf/J and PERA/EiJ; red boxes, Figure 3); their anxiety-like behavior was similar to the average behavior of the non-duplication containing strains (Figure 4E). This observation further supports the hypothesis that the presence of the duplication directly effects anxiety-like behavior.

### Relationship between the Duplication, Anxiety-like Behavior and *Glo1* expression Using Outbred CD-1 Mice

To further test the relationship between the duplication and anxiety-like behavior, we evaluated behavior in the open field in outbred CD-1 mice. We found that 52 of the 94 mice examined had the duplication while 42 did not. We also examined an additional 12 CD-1 mice that were not tested behaviorally; 7 had the duplication and 5 did not. Our PCR assay cannot discriminate between mice that are heterozygous or homozygous for the duplication; nevertheless it is possible to solve the equations p^2^+2pq+q^2^ and p+q = 1 given the data above, which yields a frequency of 0.334 for the duplication and 0.666 for the non-duplicated allele. These values apply only to the subset of 106 CD-1 mice that we genotyped, but should provide an approximate guide for future studies.

**Figure 5:**
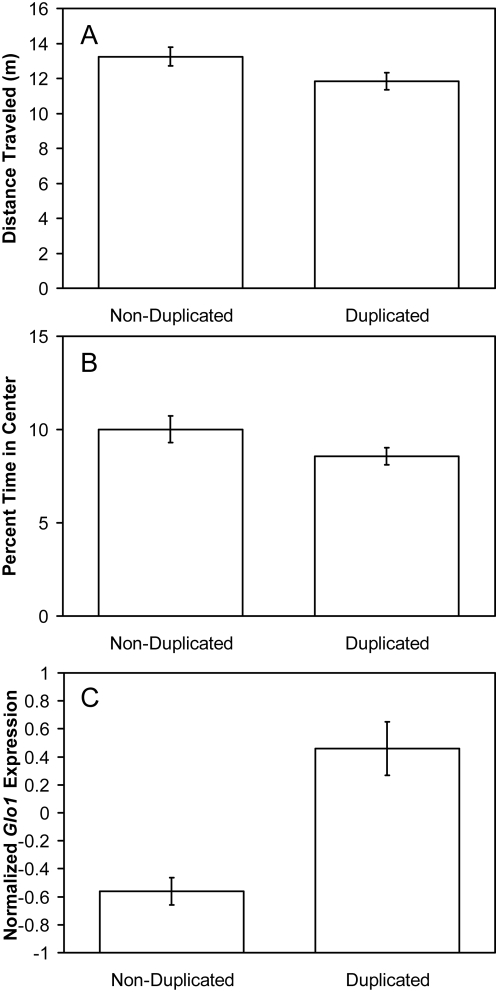
Behavior and gene expression in CD-1 mice with and without the duplication. There was significantly less activity, as measured by total distance traveled in the first 5 minutes in mice with the duplication (panel A). There was also significantly greater anxiety-like behavior, as measured as decreased percent time spent in the center of the open field in mice that had the duplication (panel B). Finally, there was a highly significant increase in *Glo1* expression in whole brain homogenates of mice with the duplication. Expression was represented as fold change versus B6 mice, which are known not to carry the duplication; values were transformed to z-scores.

We observed a significant decrease in total distance traveled in the first 5 minutes of the open field test (F(1,92) = 3.81; p<0.05; Figure 5A) and a significant decrease in the time spent in the center of the open field among CD-1 mice that were positive for the duplication (F(1,92) = 3.14; p<0.05; Figure 5B). Moreover, we observed greater *Glo1* expression among outbred CD-1 mice that were positive for the duplication (Figure 5C; F(1,57) = 19.31; p<0.00005). These data are consistent with the positive relationship between the duplication and anxiety-like behavior observed among the inbred strain panels, and support the hypothesis that these relationships are unlikely to be due to linkage between the duplication and a nearby allele. In both inbred and outbred mice many other alleles are presumed to also affect anxiety-like behavior so that the contribution of this duplication would account for only a small percentage of the total trait variance; this is characteristic of all complex traits and has made the elucidation of their molecular correlates a challenging problem.

## Discussion

Our data indicate that a CNV found in the largest extant mouse RI panel, a large inbred strain panel, an outbred laboratory mouse population and wild-caught mice causes an eQTL for *Glo1*. The CNV exists in many different haplotypes and has recently mutated to the non-duplicated state in multiple inbred strains, confounding haplotype association analysis. Furthermore, this CNV is correlated with differences in anxiety-like behavior in multiple datasets derived from studies of inbred strains and in outbred CD-1 mice. These correlations appear to persist even when the population structure of the inbred strains is taken into account. Furthermore, the relationship between *Glo1* expression and behavior does not appear to be related to linkage with alleles outside the duplication. Taken together, these data extend upon and support the observations of Hovatta et al [7] which showed a positive relationship between anxiety-like behavior and *Glo1* expression, but are inconsistent with studies based on selection for divergent levels of anxiety-like behavior in CD-1 mice, which showed a negative relationship between anxiety-like behavior and *Glo1* expression [8], [9], [10], [11]. More broadly, our data suggest that CNV are common among inbred strains and that they influence both gene expression and complex behavioral traits.

### Implications for the relationship between Glo1 expression and anxiety-like behavior

Previous data have shown a *positive* association between *Glo1* expression and anxiety-like behavior across 6 inbred strains [7]. Over- and under-expression of *Glo1* by lentiviral vectors confirmed a *positive* relationship between *Glo1* expression and anxiety-like behavior [7]. However, another study in outbred Swiss mice indicated that greater levels of anxiety-like behavior in the light dark box test were positively correlated with increases in reactive oxygen species in granulocytes [36]. Moreover, selection for anxiety-like behavior, as measured by the elevated plus maze, produced low-anxiety mice with higher *Glo1* expression, suggesting a *negative* relationship between anxiety-like behavior and *Glo1* expression and Glo1 protein levels [8], [9], [10], [11]. Thus, previous studies disagree about whether expression of *Glo1* increases or decreases anxiety-like behavior [11]. The selection studies of Kromer et al [8] used outbred CD-1 mice. Our studies have shown that CD-1 mice are polymorphic for the duplication. Therefore it seems very likely that differences in *Glo1* expression in the selected lines were due to the duplication; a combination of genetic drift and inbreeding might have caused this apparently surprising result. All data in this paper support a positive relationship between *Glo1* and anxiety-like behavior. In particular, we observed such a relationship in CD-1 mice that should be similar to those used by Kromer et al [8]. While it has been suggested that *GLO1* may be a novel target for anxiolytic intervention in humans [37], disagreement among mouse studies has been a source of major concern [11]. Our data significantly inform this debate by clearly establishing the primary molecular locus that causes differential *Glo1* expression among mice and by providing extensive support for a positive correlation between *Glo1* expression and anxiety-like behavior.

The haplotype structure shown in Figure 3 indicates that the duplication is associated with multiple distal and proximal haplotypes. We used open field data to calculate the average behavior associated with 3 of the 4 haplotypes for which data were available. This analysis showed that all duplication containing haplotypes are associated with higher anxiety-like behavior, which is consistent with a direct role for the duplication in behavior. In addition, the two strains that are part of haplotype block 2 but have lost the duplication spend more time in the center of the open field (less anxiety-like behavior) compared to strains in the same haplotype block that retain the duplication (Figure 4E). Finally, the CD-1 mice presumably have a very different haplotype structure around this duplication, but still show the expected relationship between the duplication and behavior (Figure 5). These three observations support a causal role of the duplication in behavior and are inconsistent with the alternative hypothesis that the duplication is simply in LD with some other causal allele (Figure 4E).

Because this duplication contains 4 genes it is not immediately clear which gene is responsible for the observed behavioral differences. Because 2 of the 4 genes are incomplete it is less likely that they are functional. In addition, *Glo1* was the only gene that showed a significant eQTL as a result of the duplication in any of the tissues examined. These observations suggest that *Glo1* most likely accounts for the observed behavioral differences. However the observation that over-expression or knockdown of *Glo1* altered anxiety-like behavior [7] offers the most compelling evidence that *Glo1* is responsible for the observed differences in anxiety-like behavior. In general, direct manipulation genes identified by genetic associations are critical to establish causality when multiple genes are contained within a CNV.

Thornalley [11] has suggested the association with anxiety-like behavior “…might reflect induction of *Glo1* expression as a consequence of chronic exposure to increased methylglyoxal concentration…”. Along the same lines, a recent clinical study has suggested that *GLO1* expression in blood may be a state-dependent marker of affective disorder [6]. Given that our data clearly show that differences in *Glo1* expression among mice are the direct result of this duplication, rather than the downstream result of a dynamic process, our data strongly argue that differential *Glo1* expression in mice is a trait rather than state marker of anxiety-like behavior. This further illustrates the significance of the elucidation of the molecular cause of differential *Glo1* expression described in the present report.

### Implications for other studies of *Glo1*

Beyond the relationship to anxiety-like behavior, our observations appear to explain a variety of other previously published results related to *Glo1*. In 1977, two electrophoretically distinct alleles of *Glo1* were reported to serve as genetic markers, and it was observed that an inbred female AKR/J mouse was heterozygous for this marker [38]. While the electrophoretic polymorphism is not due to the duplication, the observation of a heterozygous genotype for an inbred strain may well have been due to a polymorphism between the two duplicate copies (modern AKR/J mice have the duplication; Figure 3). A more recent study [30] suggested that differences in *Glo1* expression are caused by a mutation in *Acads*; however, in light of our data we would instead suggest that differential *Glo1* expression is due to differential fixation of the *Glo1* duplication between BALB/cByJ and BALB/cBy, and is therefore not functionally related to the *Acads* mutation (DNA from BALB/cBy and BALB/cByJ, the two strains compared by Tafti et al [30] are discordant for the duplication). Cutler et al [16] suggested that many CNVs, including the duplication of *Glp1r*, which is contained in this duplication, might be responsible for differences in food intake among inbred strains. Their analysis did not account for population structure, and may therefore overestimate the significance of this association. In addition, because *Glp1r* is only partially duplicated it is not clear that the extra copy is functional as discussed above. Thus, we believe that *Glo1* rather than *Glp1r* may be the cause of the differences observed by Cutler et al [15].

### Implications for research related to CNVs

Analysis of expression differences in the absence of knowledge of CNV can produce misleading results. No single SNP or 3-SNP haplotype was able to tag this duplication in a densely genotyped panel of inbred strains. This is due to complex haplotype structure around the duplication as well reversions to the non-duplicated state. Because many CNVs may show similar patterns, greater caution will be required when working with inbred populations because the loss of CNVs, accompanied by potentially dramatic phenotypic consequences, will be more common than has been generally appreciated [17], [18]. In addition, direct characterization of CNVs, rather than extrapolation based on shared SNP haplotypes, appears to be warranted for studies utilizing inbred strain panels. Existing recombinant inbred panels and future resources like the collaborative cross will require strategies to cope with CNVs, which cannot be correctly predicted by using nearby SNPs. Among inbred strains, sequencing a putatively duplicated region can provide a preliminary test for CNVs; the presence of heterozygous genotypes would suggest the presence of a duplication. Along the same lines, distortions of Hardy-Weinberg equilibrium would be expected for markers within a duplication when working in an outbred population. PCR assays like the one utilized in the present study can be readily developed for both deletions and duplications, providing a valuable and efficient means of genotyping large numbers of subjects. For large scale discovery and typing of copy number differences, array-based methods, such as the ones used in this paper are clearly the most efficient.

Data implicating CNV in both autism [24] and schizophrenia [25] have focused on *de novo* mutations rather than common CNV alleles. In contrast, the present study focuses on a duplication that is common among mice. In an effort to determine whether the peculiar history of inbred mouse strains had made a recent *de novo* CNV common by a combination of sampling error, genetic drift, inbreeding and perhaps unintended selection, we examined wild-caught mice (Table 1). Our studies of wild-caught mice indicated that this particular duplication is common in both wild and laboratory mice. Crosses among inbred mouse stains are efficient for identifying the effects of copy number variants that are differentially fixed among inbred strains, since the frequency of the CNV can be set at 1∶1 by crossing two contrasting inbred strains. This is a different approach than focusing on *de novo* CNV in human subjects [24], [25]; examination of *de novo* duplications shares more in common with existing mouse mutagenesis strategies. In understanding whether SNPs or CNVs found among inbred strains are the result of a rare allele that was sampled from the wild and then amplified during the inbreeding process, reference back to wild populations, as in our study, provides invaluable insights.

### Conclusions

In summary, these data show that a large CNV alters *Glo1* expression and is associated with differences in anxiety-like behavior in inbred and outbred mice. Furthermore, we show evidence that complex haplotype structure and loss of duplicated regions by non-allelic homologous recombination may degrade the statistical power of methods that seek to associate genotypes with phenotypes in panels of inbred strains. More generally, the present results show that eQTLs and genetically complex behavioral differences in mice can be caused by CNVs.

## Supporting Information

Table S1 Duplications called by the HMM relative to C57BL/6J. Table shows the beginning and end of each duplication or deletion, which strain did or did not have the duplication or deletion, as well as the size and genes involved in each feature. While the same duplication or deletion may have been identified in more than one comparison, each entry represents the boundaries as defined by a single contrast between the indicated strain and the C57BL/6J reference strain.
(0.55 MB DOC)

Table S2 Primers used to fine map and sequence the boundaries of the chromosome 17 duplication. Real time PCR primers were designed to span the predicted duplication boundaries at ∼5 kb interval. Triple lines indicate >5 kb sequence gaps in the primer survey. Primers outside the duplicated region are highlighted. ^a^ Primers used to amplify across the duplication boundary (Dup1F11/Dup1R3 or Dup1F11/Dup1R4) and to screen some inbred and outbred mice. Dup1R11, Dup1F3 or Dup1R11, Dup1F4 were simultaneously added as internal amplification controls. ^b^ Primers used for sequencing across the duplication boundary to identify the duplication boundary sequence bases. The template for the sequencing reaction was a PCR product generated using Dup1F11/Dup1R4 in B6AJF1 DNA, which was subcloned into pCR2.1 vector (Invitrogen) according to manufacturer's instructions. ^c^ Primers used to screen all strains-inbred, outbred, and wild-caught mice. Primers, qRTPDup2F/2R, span the duplication boundary. ^d^ Primers used to amplify and sequence 5′ and 3′ end of ∼1 Kb inside and flanking the duplicated region. Dup1Boundary1F/Dup1Boundary1R and qRTDup1F2/Dup1Boundary2R were used only with strains that contain the duplication. SeqBoundDup1F/Dup1SeqR3 and Dup1Boundary1F/Dup1Boundary4R were used for all strains surveyed for haplotype mapping.
(0.08 MB DOC)

Table S3 Table showing which inbred strains contain the duplication. This table indicates which strains contain a duplication of the regions on chromosome 17 from 30,174,390–30,651,226 Mb (Build 36). These data were generated by testing for the presence of the duplication using PCR primers as described in the text. DNA that was obtained from The Jackson Labs is indicated by the JAX Stock Number, DNA for strains not available from JAX were provided as gifts. Measurement of the duplication is not quantitative, in some cases more than two copies may be present for certain strains.
(0.11 MB DOC)

Table S4 Significant associations between the duplication and behaviors related to activity and emotional behaviors. Shown are all phenotypes under the JAX Phenome website defined category “behavior” that are correlated with the duplication with r≥0.5 and p≤0.05.
(0.20 MB DOC)

Figure S1 Size and frequency of duplications detected in genome-wide scan. Histograms showing frequency distribution of deletions (upper left) and duplications (upper right) as a function of size and the number of strains in which deletions (lower left) and duplications (lower left) were observed.
(5.42 MB TIF)
