# Supplementary material for: A Common and Unstable Copy Number Variant Is Associated with Differences in Glo1 Expression and Anxiety-Like Behavior
Source: PLoS One. 2009 Mar 6;4(3):e4649. doi: 10.1371/journal.pone.0004649 (PMC2650792; doi:10.1371/journal.pone.0004649)
Supplement: Table S1 — Duplications called by the HMM relative to C57BL/6J. Table shows the beginning and end of each duplication or deletion, which strain did or did not have the duplication or deletion, as well as the size and genes involved in each feature. While the same duplication or deletion may have been identified in more than one comparison, each entry represents the boundaries as defined by a single contrast between the indicated strain and the C57BL/6J reference strain. (0.55 MB DOC) [file pone.0004649.s001.doc]

**Table S1.** **Duplications called by the HMM relative to C57BL/6J.**

| Index | Start Outer | Start Inner | Stop Inner | Stop Outer | Chr | Type | Detected in | A/J | DBA/2J | LG/J | SM/J | Gene | Max Size | Min Size | # of Strains |
| --- | --- | --- | --- | --- | --- | --- | --- | --- | --- | --- | --- | --- | --- | --- | --- |
| 1 | 31034114 | 31038171 | 31060066 | 31094190 | 1 | Duplication | LG/J | absent | absent | present | absent | 4931428L18Rik, intergenic | 60076 | 21895 | 1 |
| 2 | 72324875 | 72325499 | 72376519 | 72378370 | 1 | Duplication | DBA/2J | present | present | present | present | intergenic, March4, Xrcc5 | 53495 | 51020 | 4 |
| 2 | 72324875 | 72350434 | 72376519 | 72376609 | 1 | Duplication | SM/J | present | present | present | present | intergenic, March4, Xrcc5 | 51734 | 26085 | 4 |
| 2 | 72325496 | 72326523 | 72376609 | 72378418 | 1 | Duplication | A/J | present | present | present | present | intergenic, March4, Xrcc5 | 52922 | 50086 | 4 |
| 2 | 72346846 | 72350270 | 72376519 | 72376529 | 1 | Duplication | LG/J | present | present | present | present | intergenic, March4 | 29683 | 26249 | 4 |
| 3 | 74525185 | 74525262 | 74528438 | 74528902 | 1 | Duplication | LG/J | absent | absent | present | absent | Bcs1l, intergenic, Rnf25 | 3717 | 3176 | 1 |
| 4 | 80543746 | 80544273 | 80545219 | 80545242 | 1 | Duplication | LG/J | absent | absent | present | absent | Dock10 | 1496 | 946 | 1 |
| 5 | 81761358 | 81779863 | 81837637 | 81838675 | 1 | Duplication | LG/J | absent | absent | present | absent | intergenic | 77317 | 57774 | 1 |
| 6 | 167642894 | 167646878 | 167651871 | 167661808 | 1 | Duplication | SM/J | absent | absent | absent | present | Cd247 | 18914 | 4993 | 1 |
| 7 | 190608253 | 190612547 | 190652239 | 190652368 | 1 | Duplication | DBA/2J | present | present | present | absent | Ush2a | 44115 | 39692 | 3 |
| 7 | 190617527 | 190622790 | 190652239 | 190652317 | 1 | Duplication | LG/J | present | present | present | absent | Ush2a | 34790 | 29449 | 3 |
| 7 | 190622760 | 190622806 | 190652196 | 190652317 | 1 | Duplication | A/J | present | present | present | absent | Ush2a | 29557 | 29390 | 3 |
| 8 | 93880761 | 93882816 | 93917411 | 94199345 | 3 | Duplication | DBA/2J | absent | present | present | absent | intergenic, Tdpoz4 | 318584 | 34595 | 2 |
| 8 | 93881778 | 93882816 | 94187638 | 94199346 | 3 | Duplication | LG/J | absent | present | present | absent | intergenic, Tdpoz4 | 317568 | 304822 | 2 |
| 9 | 102932501 | 102947613 | 103022622 | 103022628 | 3 | Duplication | DBA/2J | absent | present | absent | absent | intergenic, Sycp1 | 90127 | 75009 | 1 |
| 10 | 8449316 | 8488971 | 8513198 | 8521354 | 4 | Duplication | DBA/2J | absent | present | absent | absent | intergenic, Rab2 | 72038 | 24227 | 1 |
| 11 | 73835166 | 73844879 | 73854848 | 73855069 | 4 | Duplication | LG/J | absent | absent | present | absent | Jmjd2c | 19903 | 9969 | 1 |
| 12 | 130064613 | 130064926 | 130148653 | 130163457 | 4 | Duplication | A/J | present | present | present | absent | intergenic, Sdc3 | 98844 | 83727 | 3 |
| 12 | 130064613 | 130064766 | 130187884 | 130187974 | 4 | Duplication | DBA/2J | present | present | present | absent | intergenic, Laptm5, Sdc3 | 123361 | 123118 | 3 |
| 12 | 130086412 | 130088979 | 130091028 | 130094020 | 4 | Duplication | LG/J | present | present | present | absent | Sdc3 | 7608 | 2049 | 3 |
| 12 | 130097844 | 130097851 | 130111096 | 130132915 | 4 | Duplication | LG/J | present | present | present | absent | intergenic, Sdc3 | 35071 | 13245 | 3 |
| 12 | 130172991 | 130181331 | 130187601 | 130187974 | 4 | Duplication | LG/J | absent | present | present | absent | intergenic, Laptm5 | 14983 | 6270 | 2 |
| 13 | 130205710 | 130205890 | 130211687 | 130216561 | 4 | Duplication | DBA/2J | present | present | present | absent | intergenic, Laptm5, Matn1 | 10851 | 5797 | 3 |
| 13 | 130205890 | 130206546 | 130211687 | 130216681 | 4 | Duplication | A/J | present | present | present | absent | intergenic, Laptm5, Matn1 | 10791 | 5141 | 3 |
| 13 | 130206544 | 130206546 | 130211685 | 130216420 | 4 | Duplication | LG/J | present | present | present | absent | intergenic, Laptm5 | 9876 | 5139 | 3 |
| 14 | 136028695 | 136028760 | 136031251 | 136031453 | 4 | Duplication | LG/J | absent | absent | present | absent | Ephb2 | 2758 | 2491 | 1 |
| 15 | 3606082 | 3606829 | 3610819 | 3610823 | 5 | Duplication | SM/J | absent | absent | absent | present | Pex1 | 4741 | 3990 | 1 |
| 16 | 8403366 | 8403565 | 8409409 | 8409650 | 5 | Duplication | DBA/2J | present | present | absent | absent | Dbf4 | 6284 | 5844 | 2 |
| 16 | 8403595 | 8404573 | 8406184 | 8408190 | 5 | Duplication | A/J | present | present | absent | absent | Dbf4 | 4595 | 1611 | 2 |
| 17 | 78485227 | 78497630 | 78525565 | 78525568 | 6 | Duplication | LG/J | absent | absent | present | absent | intergenic | 40341 | 27935 | 1 |
| 18 | 83894444 | 83894981 | 83896180 | 83900626 | 6 | Duplication | SM/J | absent | absent | absent | present | intergenic | 6182 | 1199 | 1 |
| 19 | 85242767 | 85243303 | 85247069 | 85247537 | 6 | Duplication | LG/J | absent | absent | present | absent | Sfxn5 | 4770 | 3766 | 1 |
| 20 | 126583214 | 126585182 | 126591994 | 126594134 | 6 | Duplication | LG/J | absent | absent | present | absent | intergenic | 10920 | 6812 | 1 |
| 21 | 141371291 | 141371959 | 141374826 | 141378036 | 6 | Duplication | LG/J | absent | absent | present | absent | Pde3a | 6745 | 2867 | 1 |
| 22 | 143129658 | 143134024 | 143138039 | 143141885 | 6 | Duplication | SM/J | absent | absent | absent | present | Etnk1 | 12227 | 4015 | 1 |
| 23 | 144358407 | 144359348 | 144359757 | 144362359 | 6 | Duplication | LG/J | absent | absent | present | absent | intergenic | 3952 | 409 | 1 |
| 24 | 46159043 | 46163468 | 46166337 | 46166640 | 7 | Duplication | SM/J | absent | absent | absent | present | Otog | 7597 | 2869 | 1 |
| 25 | 49027712 | 49027792 | 49031250 | 49031250 | 7 | Duplication | SM/J | absent | absent | absent | present | intergenic | 3538 | 3458 | 1 |
| 26 | 80965967 | 80967016 | 81034574 | 81034777 | 7 | Duplication | A/J | present | absent | absent | absent | Alpk3, intergenic, Slc28a1 | 68810 | 67558 | 1 |
| 27 | 105525142 | 105525627 | 105542945 | 105547738 | 7 | Duplication | A/J | present | absent | absent | absent | 8030491N06Rik, intergenic | 22596 | 17318 | 1 |
| 28 | 140713187 | 140713945 | 140714319 | 140714378 | 7 | Duplication | SM/J | absent | absent | absent | present | Ric8 | 1191 | 374 | 1 |
| 29 | 75249930 | 75250061 | 75260792 | 75260813 | 8 | Duplication | LG/J | absent | absent | present | absent | intergenic, Klf2 | 10883 | 10731 | 1 |
| 30 | 77921998 | 77926075 | 77927969 | 77929255 | 8 | Duplication | LG/J | absent | absent | present | absent | Hmgb2l1, intergenic | 7257 | 1894 | 1 |
| 31 | 125060155 | 125060564 | 125087168 | 125098581 | 8 | Duplication | SM/J | absent | absent | absent | present | intergenic | 38426 | 26604 | 1 |
| 32 | 3015282 | 3015282 | 3062573 | 3077502 | 9 | Duplication | DBA/2J | absent | present | absent | absent | intergenic | 62220 | 47291 | 1 |
| 33 | 108820989 | 108821159 | 108913714 | 108913747 | 9 | Duplication | LG/J | absent | absent | present | absent | Col7a1, intergenic, Pfkfb4, Scotin | 92758 | 92555 | 1 |
| 34 | 75592831 | 75602306 | 75649187 | 75650963 | 11 | Duplication | SM/J | absent | absent | absent | present | Doc2b, intergenic, Rph3al | 58132 | 46881 | 1 |
| 35 | 78341556 | 78343534 | 78346302 | 78346439 | 11 | Duplication | LG/J | absent | absent | present | absent | Tnfaip1 | 4883 | 2768 | 1 |
| 36 | 90465470 | 90465702 | 90466829 | 90468021 | 11 | Duplication | LG/J | absent | absent | present | absent | intergenic | 2551 | 1127 | 1 |
| 37 | 67360680 | 67360688 | 67970141 | 67981739 | 12 | Duplication | LG/J | absent | absent | present | absent | intergenic, Mamdc1 | 621059 | 609453 | 1 |
| 38 | 116015068 | 116040999 | 116102737 | 116112922 | 12 | Duplication | SM/J | absent | absent | absent | present | intergenic, LOC380824, LOC435328, LOC636462, LOC668563 | 97854 | 61738 | 1 |
| 39 | 95201981 | 95202220 | 95205877 | 95207414 | 13 | Duplication | LG/J | absent | absent | present | absent | Lhfpl2 | 5433 | 3657 | 1 |
| 40 | 104319089 | 104331930 | 104351942 | 104353269 | 13 | Duplication | LG/J | absent | absent | present | absent | Mast4 | 34180 | 20012 | 1 |
| 41 | 38763913 | 38764009 | 38765348 | 38765382 | 15 | Duplication | SM/J | absent | absent | absent | present | Baalc, intergenic | 1469 | 1339 | 1 |
| 42 | 98136299 | 98140912 | 98141120 | 98158881 | 15 | Duplication | DBA/2J | absent | present | absent | absent | intergenic, Olfr285 | 22582 | 208 | 1 |
| 43 | 3150625 | 3150625 | 3161861 | 3164618 | 16 | Duplication | A/J | present | absent | absent | absent | intergenic | 13993 | 11236 | 1 |
| 44 | 8304754 | 8309699 | 8368243 | 8368267 | 17 | Duplication | DBA/2J | present | present | present | absent | intergenic | 63513 | 58544 | 3 |
| 44 | 8305966 | 8309699 | 8365038 | 8368243 | 17 | Duplication | A/J | present | present | present | absent | intergenic | 62277 | 55339 | 3 |
| 44 | 8305977 | 8318604 | 8365029 | 8365199 | 17 | Duplication | LG/J | present | present | present | absent | intergenic | 59222 | 46425 | 3 |
| 45 | 8461724 | 8461994 | 8688357 | 8688906 | 17 | Duplication | A/J | present | present | present | absent | intergenic, Pde10a | 227182 | 226363 | 3 |
| 45 | 8464629 | 8465722 | 8688633 | 8688906 | 17 | Duplication | DBA/2J | present | present | present | absent | intergenic, Pde10a | 224277 | 222911 | 3 |
| 45 | 8568919 | 8606727 | 8667564 | 8688633 | 17 | Duplication | LG/J | present | present | present | absent | intergenic, Pde10a | 119714 | 60837 | 3 |
| 46 | 20946749 | 20966073 | 20991319 | 20991327 | 17 | Duplication | A/J | present | absent | absent | absent | intergenic, V1rf2, V1rf4 | 44578 | 25246 | 1 |
| 47 | 30173626 | 30174462 | 30652623 | 30656950 | 17 | Duplication | A/J | present | present | absent | absent | Btbd9, Dnahc8, Glo1, Glp1r, intergenic | 483324 | 478161 | 2 |
| 47 | 30174557 | 30185839 | 30553943 | 30581833 | 17 | Duplication | DBA/2J | present | present | absent | absent | Btbd9, Dnahc8, Glo1, intergenic | 407276 | 368104 | 2 |
| 47 | 30602003 | 30614197 | 30628845 | 30633376 | 17 | Duplication | DBA/2J | present | present | absent | absent | Dnahc8, Glp1r, intergenic | 31373 | 14648 | 2 |
| 48 | 30973884 | 30976650 | 30996899 | 31005981 | 17 | Duplication | LG/J | absent | absent | present | absent | intergenic, Tsga2 | 32097 | 20249 | 1 |
| 49 | 34857017 | 34857957 | 34871630 | 34874265 | 17 | Duplication | A/J | present | present | absent | absent | Atp6v1g2, H2-Q5, intergenic | 17248 | 13673 | 2 |
| 49 | 34857084 | 34857590 | 34871481 | 34873016 | 17 | Duplication | DBA/2J | present | present | absent | absent | Atp6v1g2, H2-Q5, intergenic | 15932 | 13891 | 2 |
| 50 | 35081982 | 35082025 | 35094010 | 35096982 | 17 | Duplication | SM/J | absent | absent | absent | present | H2-Q10, intergenic | 15000 | 11985 | 1 |
| 51 | 38086016 | 38163882 | 38189231 | 38249144 | 17 | Duplication | DBA/2J | absent | present | present | absent | intergenic | 163128 | 25349 | 2 |
| 51 | 38086178 | 38163882 | 38189098 | 38189231 | 17 | Duplication | LG/J | absent | present | present | absent | intergenic | 103053 | 25216 | 2 |
| 52 | 39416429 | 39451281 | 39472696 | 39535808 | 17 | Duplication | A/J | present | absent | absent | absent | intergenic | 119379 | 21415 | 1 |
| 53 | 55903719 | 55903753 | 55904067 | 55904440 | 17 | Duplication | SM/J | absent | absent | absent | present | Ticam1 | 721 | 314 | 1 |
| 54 | 56013133 | 56013257 | 56015054 | 56016880 | 17 | Duplication | SM/J | absent | absent | absent | present | Jmjd2b | 3747 | 1797 | 1 |
| 55 | 15863772 | 15866050 | 15871775 | 15878851 | 19 | Duplication | SM/J | absent | absent | absent | present | intergenic | 15079 | 5725 | 1 |
| 56 | 46365128 | 46365180 | 46365442 | 46365593 | 19 | Duplication | SM/J | absent | absent | absent | present | Nfkb2, Psd | 465 | 262 | 1 |
| 57 | 3052223 | 3052223 | 4293543 | 4442999 | X | Duplication | A/J | present | absent | present | absent | intergenic | 1390776 | 1241320 | 2 |
| 57 | 3052223 | 3052223 | 4293548 | 4532217 | X | Duplication | LG/J | present | absent | present | absent | intergenic | 1479994 | 1241325 | 2 |
| 58 | 6752030 | 6752084 | 6752309 | 6752512 | X | Duplication | SM/J | absent | absent | absent | present | Ccdc22, Foxp3 | 482 | 225 | 1 |
| 59 | 21430398 | 21430419 | 21462982 | 21462984 | X | Duplication | SM/J | absent | absent | absent | present | intergenic | 32586 | 32563 | 1 |
| 60 | 32323023 | 32331238 | 32335738 | 32335738 | X | Duplication | SM/J | absent | absent | absent | present | Dock11 | 12715 | 4500 | 1 |
| 61 | 52909713 | 52909757 | 52918808 | 52918823 | X | Duplication | SM/J | absent | absent | absent | present | intergenic | 9110 | 9051 | 1 |
| 62 | 67379920 | 67381415 | 67381929 | 67381993 | X | Duplication | SM/J | absent | absent | absent | present | G630014P10Rik | 2073 | 514 | 1 |
| 63 | 102636297 | 102640512 | 102651945 | 102651970 | X | Duplication | LG/J | absent | absent | present | absent | Gm784 | 15673 | 11433 | 1 |
| 64 | 102676639 | 102686332 | 102698562 | 102747559 | X | Duplication | LG/J | absent | absent | present | absent | Gm784, intergenic | 70920 | 12230 | 1 |
| 65 | 116407341 | 116407860 | 116407894 | 116408202 | X | Duplication | SM/J | absent | absent | absent | present | intergenic | 861 | 34 | 1 |
| 66 | 147958039 | 147958352 | 147958627 | 147959866 | X | Duplication | SM/J | present | absent | absent | present | Apxl | 1827 | 275 | 2 |
| 66 | 147958084 | 147958314 | 147958729 | 147984237 | X | Duplication | A/J | present | absent | absent | present | Apxl | 26153 | 415 | 2 |
| 67 | 152686811 | 152686823 | 152689737 | 152689763 | X | Duplication | SM/J | absent | absent | absent | present | Phex | 2952 | 2914 | 1 |
| 68 | 158376459 | 158376482 | 158377688 | 158378180 | X | Duplication | SM/J | absent | absent | absent | present | intergenic | 1721 | 1206 | 1 |
| 69 | 24710722 | 24710722 | 24720057 | 24720063 | 1 | Deletion | SM/J | absent | absent | absent | present | intergenic | 9341 | 9335 | 1 |
| 70 | 38162958 | 38162977 | 38168866 | 38168866 | 1 | Deletion | LG/J | absent | absent | present | absent | Aff3 | 5908 | 5889 | 1 |
| 71 | 38184447 | 38184515 | 38187506 | 38189734 | 1 | Deletion | LG/J | absent | absent | present | absent | Aff3 | 5287 | 2991 | 1 |
| 72 | 38191038 | 38191056 | 38193728 | 38193728 | 1 | Deletion | LG/J | absent | absent | present | absent | Aff3 | 2690 | 2672 | 1 |
| 73 | 38219226 | 38219231 | 38223498 | 38223499 | 1 | Deletion | LG/J | absent | absent | present | absent | Aff3 | 4273 | 4267 | 1 |
| 74 | 66647841 | 66647841 | 66649184 | 66649191 | 1 | Deletion | SM/J | absent | absent | absent | present | Rpe | 1350 | 1343 | 1 |
| 75 | 98754700 | 98754701 | 98777268 | 98777268 | 1 | Deletion | LG/J | absent | absent | present | absent | Slco6b1 | 22568 | 22567 | 1 |
| 76 | 112973882 | 112973964 | 112998954 | 112998954 | 1 | Deletion | LG/J | absent | absent | present | present | intergenic | 25072 | 24990 | 2 |
| 76 | 112973882 | 112973964 | 112998954 | 112998954 | 1 | Deletion | SM/J | absent | absent | present | present | intergenic | 25072 | 24990 | 2 |
| 77 | 113074414 | 113107108 | 113174816 | 113175082 | 1 | Deletion | LG/J | absent | absent | present | present | intergenic | 100668 | 67708 | 2 |
| 77 | 113074414 | 113107108 | 113174811 | 113175082 | 1 | Deletion | SM/J | absent | absent | present | present | intergenic | 100668 | 67703 | 2 |
| 78 | 113213534 | 113213599 | 113385250 | 113385547 | 1 | Deletion | SM/J | absent | present | present | present | intergenic | 172013 | 171651 | 3 |
| 78 | 113226270 | 113226270 | 113334839 | 113385235 | 1 | Deletion | LG/J | absent | present | present | present | intergenic | 158965 | 108569 | 3 |
| 78 | 113226301 | 113251914 | 113266917 | 113274197 | 1 | Deletion | DBA/2J | absent | present | present | present | intergenic | 47896 | 15003 | 3 |
| 79 | 113468290 | 113468296 | 113486487 | 113488466 | 1 | Deletion | LG/J | absent | absent | present | present | intergenic | 20176 | 18191 | 2 |
| 79 | 113468296 | 113468296 | 113488466 | 113488466 | 1 | Deletion | SM/J | absent | absent | present | present | intergenic | 20170 | 20170 | 2 |
| 80 | 114180389 | 114190738 | 114214227 | 114214235 | 1 | Deletion | SM/J | absent | absent | present | present | intergenic | 33846 | 23489 | 2 |
| 80 | 114190684 | 114190694 | 114209922 | 114214227 | 1 | Deletion | LG/J | absent | absent | present | present | intergenic | 23543 | 19228 | 2 |
| 81 | 114230636 | 114232283 | 114233709 | 114242255 | 1 | Deletion | LG/J | absent | absent | present | present | intergenic | 11619 | 1426 | 2 |
| 81 | 114230648 | 114232283 | 114233692 | 114233692 | 1 | Deletion | SM/J | absent | absent | present | present | intergenic | 3044 | 1409 | 2 |
| 82 | 114282263 | 114282266 | 114283102 | 114283704 | 1 | Deletion | LG/J | absent | absent | present | present | intergenic | 1441 | 836 | 2 |
| 82 | 114282263 | 114282266 | 114283704 | 114283704 | 1 | Deletion | SM/J | absent | absent | present | present | intergenic | 1441 | 1438 | 2 |
| 83 | 114319959 | 114319959 | 114431520 | 114431520 | 1 | Deletion | LG/J | absent | absent | present | present | intergenic | 111561 | 111561 | 2 |
| 83 | 114319959 | 114319959 | 114431520 | 114431520 | 1 | Deletion | SM/J | absent | absent | present | present | intergenic | 111561 | 111561 | 2 |
| 84 | 133853711 | 133853728 | 133853931 | 133854793 | 1 | Deletion | LG/J | absent | absent | present | present | intergenic | 1082 | 203 | 2 |
| 84 | 133853728 | 133853728 | 133854387 | 133854434 | 1 | Deletion | SM/J | absent | absent | present | present | intergenic | 706 | 659 | 2 |
| 85 | 159256814 | 159256814 | 159260664 | 159260674 | 1 | Deletion | SM/J | absent | absent | absent | present | 2810025M15Rik, intergenic | 3860 | 3850 | 1 |
| 86 | 35002278 | 35002348 | 35002394 | 35002402 | 2 | Deletion | LG/J | absent | absent | present | absent | Rab14 | 124 | 46 | 1 |
| 87 | 48065104 | 48066163 | 48112087 | 48112094 | 3 | Deletion | DBA/2J | absent | present | absent | absent | intergenic | 46990 | 45924 | 1 |
| 88 | 48231479 | 48231479 | 48250698 | 48291097 | 3 | Deletion | SM/J | absent | absent | absent | present | intergenic | 59618 | 19219 | 1 |
| 89 | 3006643 | 3006643 | 3006963 | 3007129 | 4 | Deletion | A/J | present | present | present | present | intergenic | 486 | 320 | 4 |
| 89 | 3006643 | 3006643 | 3009068 | 3009092 | 4 | Deletion | DBA/2J | present | present | present | present | intergenic | 2449 | 2425 | 4 |
| 89 | 3006643 | 3006643 | 3007129 | 3007224 | 4 | Deletion | LG/J | present | present | present | present | intergenic | 581 | 486 | 4 |
| 89 | 3006643 | 3006643 | 3007129 | 3007133 | 4 | Deletion | SM/J | present | present | present | present | intergenic | 490 | 486 | 4 |
| 89 | 3007531 | 3007841 | 3009068 | 3009068 | 4 | Deletion | LG/J | absent | present | present | present | intergenic | 1537 | 1227 | 3 |
| 89 | 3007709 | 3007841 | 3009068 | 3009068 | 4 | Deletion | SM/J | absent | present | present | present | intergenic | 1359 | 1227 | 3 |
| 90 | 147412234 | 147412452 | 147413556 | 147414202 | 4 | Deletion | LG/J | absent | absent | present | absent | Exosc10 | 1968 | 1104 | 1 |
| 91 | 44471613 | 44471633 | 44472491 | 44472491 | 5 | Deletion | LG/J | absent | absent | present | absent | 4932414K18Rik | 878 | 858 | 1 |
| 92 | 122950582 | 122951933 | 122954264 | 122957016 | 5 | Deletion | A/J | present | absent | present | present | intergenic, P2rx7 | 6434 | 2331 | 3 |
| 92 | 122950582 | 122951959 | 122954253 | 122954258 | 5 | Deletion | LG/J | present | absent | present | present | intergenic, P2rx7 | 3676 | 2294 | 3 |
| 92 | 122951891 | 122951959 | 122952831 | 122954253 | 5 | Deletion | SM/J | present | absent | present | present | intergenic, P2rx7 | 2362 | 872 | 3 |
| 93 | 25455114 | 25455115 | 25459125 | 25459173 | 7 | Deletion | SM/J | absent | absent | absent | present | Hnrpul1 | 4059 | 4010 | 1 |
| 94 | 109519035 | 109519047 | 109519170 | 109519182 | 7 | Deletion | SM/J | absent | absent | absent | present | Ascl3 | 147 | 123 | 1 |
| 95 | 113952965 | 113952976 | 113953080 | 113954029 | 7 | Deletion | SM/J | absent | absent | absent | present | intergenic | 1064 | 104 | 1 |
| 96 | 132394469 | 132394472 | 132396438 | 132396469 | 7 | Deletion | SM/J | absent | absent | absent | present | intergenic, Oat | 2000 | 1966 | 1 |
| 97 | 62944963 | 62944963 | 62946004 | 62946017 | 9 | Deletion | LG/J | absent | absent | present | absent | intergenic, Lbxcor1 | 1054 | 1041 | 1 |
| 98 | 38677894 | 38677894 | 38681210 | 38681210 | 10 | Deletion | SM/J | absent | absent | absent | present | Lama4 | 3316 | 3316 | 1 |
| 99 | 51444886 | 51444886 | 51445027 | 51445034 | 10 | Deletion | LG/J | absent | absent | present | absent | 4933411G06Rik | 148 | 141 | 1 |
| 100 | 3034629 | 3034629 | 3039637 | 3039637 | 11 | Deletion | LG/J | absent | absent | present | absent | Sfi1 | 5008 | 5008 | 1 |
| 101 | 3044872 | 3044990 | 3048891 | 3050043 | 11 | Deletion | LG/J | absent | absent | present | present | intergenic, Sfi1 | 5171 | 3901 | 2 |
| 101 | 3044872 | 3044959 | 3097041 | 3101800 | 11 | Deletion | SM/J | absent | absent | present | present | intergenic, Sfi1 | 56928 | 52082 | 2 |
| 101 | 3051364 | 3052128 | 3057674 | 3059356 | 11 | Deletion | LG/J | absent | absent | present | present | intergenic | 7992 | 5546 | 2 |
| 101 | 3061374 | 3061533 | 3093256 | 3093256 | 11 | Deletion | LG/J | absent | absent | present | present | intergenic | 31882 | 31723 | 2 |
| 101 | 3093502 | 3093595 | 3097041 | 3097041 | 11 | Deletion | LG/J | absent | absent | present | present | intergenic | 3539 | 3446 | 2 |
| 102 | 5659147 | 5660423 | 5661733 | 5661733 | 11 | Deletion | LG/J | absent | absent | present | present | intergenic | 2586 | 1310 | 2 |
| 102 | 5659147 | 5660423 | 5661733 | 5661773 | 11 | Deletion | SM/J | absent | absent | present | present | intergenic | 2626 | 1310 | 2 |
| 103 | 104699232 | 104709802 | 104716693 | 104716693 | 11 | Deletion | DBA/2J | absent | present | absent | absent | intergenic | 17461 | 6891 | 1 |
| 104 | 20933691 | 20964579 | 20986424 | 20986621 | 12 | Deletion | DBA/2J | absent | present | absent | absent | LOC668621 | 52930 | 21845 | 1 |
| 105 | 57444377 | 57445101 | 57449994 | 57449994 | 13 | Deletion | SM/J | absent | absent | absent | present | Spock1 | 5617 | 4893 | 1 |
| 106 | 35861019 | 35865222 | 35869605 | 35869746 | 14 | Deletion | LG/J | absent | absent | present | absent | Gcap14 | 8727 | 4383 | 1 |
| 107 | 40128452 | 40240515 | 41937924 | 42412430 | 14 | Deletion | DBA/2J | present | present | absent | absent | 1700001F09Rik, 1700024B05Rik, intergenic, LOC638353, LOC666099, LOC666207, LOC666285, LOC666415, LOC666471, LOC666485 | 2283978 | 1697409 | 2 |
| 107 | 42168941 | 42168941 | 42412430 | 42412430 | 14 | Deletion | A/J | present | present | absent | absent | 1700001F09Rik, intergenic, LOC666485 | 243489 | 243489 | 2 |
| 108 | 43443412 | 43452977 | 43547053 | 43612966 | 14 | Deletion | DBA/2J | absent | present | absent | absent | intergenic, LOC624927, LOC666692, LOC666699, LOC666723 | 169554 | 94076 | 1 |
| 109 | 50481227 | 50492767 | 50584162 | 50584163 | 14 | Deletion | DBA/2J | absent | present | absent | absent | LOC434459, LOC638695 | 102936 | 91395 | 1 |
| 110 | 68375768 | 68379771 | 68379977 | 68383247 | 14 | Deletion | LG/J | present | absent | present | absent | Loxl2 | 7479 | 206 | 2 |
| 110 | 68379779 | 68379779 | 68383247 | 68383247 | 14 | Deletion | A/J | present | absent | present | absent | Loxl2 | 3468 | 3468 | 2 |
| 111 | 40247532 | 40258107 | 40265689 | 40265690 | 19 | Deletion | A/J | present | absent | present | absent | intergenic, LOC668299 | 18158 | 7582 | 2 |
| 111 | 40247610 | 40258107 | 40265689 | 40265698 | 19 | Deletion | LG/J | present | absent | present | absent | intergenic, LOC668299 | 18088 | 7582 | 2 |
| 112 | 102640512 | 102640513 | 102814799 | 102844271 | X | Deletion | A/J | present | present | absent | present | Cysltr1, Gm784, intergenic, LOC622527 | 203759 | 174286 | 3 |
| 112 | 102640512 | 102640513 | 102675865 | 102675865 | X | Deletion | DBA/2J | present | present | absent | present | Gm784 | 35353 | 35352 | 3 |
| 112 | 102640513 | 102648387 | 102845052 | 102845052 | X | Deletion | SM/J | present | present | absent | present | Cysltr1, Gm784, intergenic, LOC622527 | 204539 | 196665 | 3 |
| 112 | 102688198 | 102688353 | 102790979 | 102805978 | X | Deletion | DBA/2J | present | present | absent | present | Cysltr1, intergenic | 117780 | 102626 | 3 |

Table shows the beginning and end of each duplication or deletion, which strain did or did not have the duplication or deletion, as well as the size and genes involved in each feature. While the same duplication or deletion may have been identified in more than one comparison, each entry represents the boundaries as defined by a single contrast between the indicated strain and the C57BL/6J reference strain.
