# Supplementary material for: A Common and Unstable Copy Number Variant Is Associated with Differences in Glo1 Expression and Anxiety-Like Behavior
Source: PLoS One. 2009 Mar 6;4(3):e4649. doi: 10.1371/journal.pone.0004649 (PMC2650792; doi:10.1371/journal.pone.0004649)
Supplement: Table S2 — Primers used to fine map and sequence the boundaries of the chromosome 17 duplication. Real time PCR primers were designed to span the predicted duplication boundaries at ∼5 kb interval. Triple lines indicate >5 kb sequence gaps in the primer survey. Primers outside the duplicated region are highlighted. a Primers used to amplify across the duplication boundary (Dup1F11/Dup1R3 or Dup1F11/Dup1R4) and to screen some inbred and outbred mice. Dup1R11, Dup1F3 or Dup1R11, Dup1F4 were simultaneously added as internal amplification controls. b Primers used for sequencing across the duplication boundary to identify the duplication boundary sequence bases. The template for the sequencing reaction was a PCR product generated using Dup1F11/Dup1R4 in B6AJF1 DNA, which was subcloned into pCR2.1 vector (Invitrogen) according to manufacturer's instructions. c Primers used to screen all strains-inbred, outbred, and wild-caught mice. Primers, qRTPDup2F/2R, span the duplication boundary. d Primers used to amplify and sequence 5′ and 3′ end of ∼1 Kb inside and flanking the duplicated region. Dup1Boundary1F/Dup1Boundary1R and qRTDup1F2/Dup1Boundary2R were used only with strains that contain the duplication. SeqBoundDup1F/Dup1SeqR3 and Dup1Boundary1F/Dup1Boundary4R were used for all strains surveyed for haplotype mapping. (0.08 MB DOC) [file pone.0004649.s002.doc]

**Table S2** -- **Primers used to fine map and sequence the boundaries of the chromosome 17 duplication.**

| Primer Name | Primer Sequence | Position |
| --- | --- | --- |
| Dup1F1 | AAAAGTTCCCTTACAGACTCCTGTT | 30167662 30167686 |
| Dup1R1 | AAAGGGAAATAGTCCAGAGTTCAGT | 30168290 30168314 |
| Dup1F2 | TGAGCCAACAGTGAGATAACAACTC | 30172985 30173009 |
| Dup1R2 | CTTGGTGTTTCTTCAGACTGTCATG | 30173589 30173613 |
| Dup1F3^a^ | TAAGGCTTGCCATGGTGTCCGTTCA | 30177575 30177599 |
| Dup1R3^a^ | GGCCAGGTCAACACATTACTCCCAG | 30177781 30177805 |
| Dup1F4^a^ | CAGTGGCTGGCCTTCTGCAGTAGAA | 30182581 30182605 |
| Dup1R4^a^ | CAATTTTCAGTCGTCAGAGGGGCCA | 30183313 30183337 |
| Dup1F6 | CAGATGTTGCTAAGTCCCATGGTGA | 30625273 30625297 |
| Dup1R6 | TCAGTGTGCATCACCCTGGGACTTG | 30625391 30625415 |
| Dup1F7 | ATTTGCAGATTAGGTTGAGAGTGAG | 30630288 30630312 |
| Dup1R7 | GAAGGTGATCTCACAGACAGAAAGT | 30630635 30630659 |
| Dup1F8 | CTTTGATGGAGTCTTCTCTTGACTC | 30634679 30634703 |
| Dup1R8 | TGCCATCTAGTAAGTACCCAGAGAG | 30634914 30634938 |
| Dup1F10 | ACAAGGCCATGTGTACCGGTTCTGC | 30645896 30645920 |
| Dup1R10 | AAGGATGGCTGAAGCGATGACCAAG | 30646439 30646463 |
| Dup1F11^a^ | TAGAATGTGTTAGGGAACCCACTGC | 30650251 30650275 |
| Dup1R11^a^ | CAACTGTATGGCAAACTGGGAATCC | 30650522 30650546 |
| Dup1F12 | CCAGGGACAGACCATTAAGGAACTC | 30653774 30653798 |
| Dup1R12 | TCTGAGCCCTGAGCAAGGATAGGCA | 30654010 30654034 |
| Dup1SeqF1^b^ | TGCTGCATGTACACCCAAAT | 30650774 30650793 |
| Dup1SeqF2^b^ | CTCTGCCCCAGAGAACAGTC | 30651154 30651173 |
| Dup1SeqF3^b^ | ATCCACCTGAACCTGTTTGC | 30651538 30651557 |
| Dup1SeqF5^b^ | CTGCTGTGTGGCCTCTATCA | 30174442 30174461 |
| Dup1SeqR2^b^ | GGCCTAGCTGCAGAATTGTC | 30174410 30174429 |
| Dup1SeqR3^b,d^ | GAGCTGAAGGGATCTGCAAC | 30175124 30175144 |
| qRTDup1F1^b,c^ | TGCTGCATGTACACCCAAAT | 30650774 30650793 |
| qRTDup1R1^b,c^ | ACAGAAGACCACTGGGATGG | 30650923 30650942 |
| qRTDup1F2^b,c,d^ | CTCTGCCCCAGAGAACAGTC | 30651154 30651173 |
| qRTDup1R2^b,c^ | TGATAGAGGCCACACAGCAG | 30174442 30174461 |
| Dup1Boundary1F^d^ | TGCTTGGGAGGTAGCCTTTA | 30650485 30650504 |
| Dup1Boundary1R^d^ | GCAGCACTGAGGGTTTGTCT | 30174562 30174581 |
| Dup1Boundary2R^d^ | GACCCTGGTATAGCTGTCTCGTAT | 30175348 30175373 |
| SeqBoundDup1F^d^ | CAGTCGTCGACAGTCATCGT | 30174226 30174245 |
| Dup1Boundary4R^d^ | AGGCATGTATCCACCTCTGG | 30651398 30651417 |

Real time PCR primers were designed to span the predicted duplication boundaries at ~5kb interval. Triple lines indicate >5kb sequence gaps in the primer survey. Primers outside the duplicated region are highlighted.

^a^ Primers used to amplify across the duplication boundary (Dup1F11/Dup1R3 or Dup1F11/Dup1R4) and to screen some inbred and outbred mice. Dup1R11, Dup1F3 or Dup1R11, Dup1F4 were simultaneously added as internal amplification controls.

^b^Primers used for sequencing across the duplication boundary to identify the duplication boundary sequence bases. The template for the sequencing reaction was a PCR product generated using Dup1F11/Dup1R4 in B6AJF1 DNA, which was subcloned into pCR2.1 vector (Invitrogen) according to manufacturer’s instructions.

^c^Primers used to screen all strains—inbred, outbred, and wild-caught mice. Primers, qRTPDup2F/2R, span the duplication boundary.

^d^Primers used to amplify and sequence 5’ and 3’ end of ~1 Kb inside and flanking the duplicated region. Dup1Boundary1F/Dup1Boundary1R and qRTDup1F2/Dup1Boundary2R were used only with strains that contain the duplication. SeqBoundDup1F/Dup1SeqR3 and Dup1Boundary1F/Dup1Boundary4R were used for all strains surveyed for haplotype mapping.
