# Supplementary material for: A Common and Unstable Copy Number Variant Is Associated with Differences in Glo1 Expression and Anxiety-Like Behavior
Source: PLoS One. 2009 Mar 6;4(3):e4649. doi: 10.1371/journal.pone.0004649 (PMC2650792; doi:10.1371/journal.pone.0004649)
Supplement: Table S3 — Table showing which inbred strains contain the duplication. This table indicates which strains contain a duplication of the regions on chromosome 17 from 30,174,390–30,651,226 Mb (Build 36). These data were generated by testing for the presence of the duplication using PCR primers as described in the text. DNA that was obtained from The Jackson Labs is indicated by the JAX Stock Number, DNA for strains not available from JAX were provided as gifts. Measurement of the duplication is not quantitative, in some cases more than two copies may be present for certain strains. (0.11 MB DOC) [file pone.0004649.s003.doc]

**Table S3** -- **Table showing which inbred strains contain the duplication.**

| **Strain Name** | **JAX Stock Number** | **Duplication (0=absent; 1=present)** |
| --- | --- | --- |
| 129S1/SvImJ | 2448 | 1 |
| 129X1/SvJ | 691 | 0 |
| A/J | 646 | 1 |
| AKR/J | 648 | 1 |
| BALB/cByJ | 1026 | 1 |
| BALB/cJ | 651 | 0 |
| BPH/2@J | 3005 | 1 |
| BPL/1@J | 3006 | 0 |
| BPN/3@J | 3004 | 1 |
| BTBR T+ tf/J | 2282 | 0 |
| BUB/BnJ | 653 | 0 |
| C3H/HeJ | 659 | 1 |
| C57BL/10J | 665 | 0 |
| C57BL/6J | 664 | 0 |
| C57BLKS/J | 662 | 1 |
| C57BR/cdJ | 667 | 0 |
| C57L/J | 668 | 0 |
| C58/J | 669 | 0 |
| CALB/RkJ | 1489 | 0 |
| CAST/EiJ | 928 | 0 |
| CBA/J | 656 | 1 |
| CE/J | 657 | 1 |
| CZECHII/EiJ | 1144 | 0 |
| DBA/1J | 670 | 1 |
| DBA/2J | 671 | 1 |
| DDK/Pas | - | 0 |
| DDY/JclSidSeyFrkJ | 2243 | 0 |
| El/SuzSeyFrkJ | 1956 | 0 |
| FVB/NJ | 1800 | 0 |
| HTG/GoSfSnJ | 0556 | 1 |
| I/LnJ | 674 | 1 |
| ILS | - | 1 |
| IS/CamRkJ | 573 | 1 |
| ISS | - | 1 |
| JF1/Ms | 3720 | 0 |
| KK/HlJ | 2106 | 0 |
| LEWES/EiJ | 2798 | 1 |
| LG/J | 675 | 0 |
| LP/J | 676 | 1 |
| MA/MyJ | 677 | 0 |
| MAI/Pas | - | 0 |
| MOLF/EiJ | 550 | 0 |
| MOLG/DnJ | 555 | 0 |
| MRL/MpJ | 486 | 1 |
| MSM/Ms | 3719 | 0 |
| NOD/ShiLtJ | 1976 | 0 |
| NON/ShiLtJ | 2423 | 0 |
| NOR/LtJ | 2050 | 0 |
| NZB/BlNJ | 684 | 0 |
| NZO/HILtJ | 2105 | 0 |
| NZW/LacJ | 1058 | 0 |
| P/J | 679 | 1 |
| PERA/EiJ | 930 | 0 |
| PERC/EiJ | 1307 | 1 |
| PL/J | 680 | 0 |
| PWK/PhJ | 3715 | 0 |
| RBF/DnJ | 726 | 0 |
| RF/J | 682 | 0 |
| RIIIS/J | 683 | 0 |
| SEA/GnJ | 644 | 1 |
| SEG/Pas | - | 0 |
| SJL/J | 686 | 0 |
| SKIVE/EiJ | 1393 | 0 |
| SM/J | 687 | 0 |
| SOD1/EiJ | 1224 | 0 |
| SPRET/EiJ | 1146 | 0 |
| ST/bJ | 688 | 0 |
| SWR/J | 689 | 0 |
| TALLYHO/JngJ | 5314 | 0 |
| WSB/EiJ | 1145 | 0 |
| ZALENDE/EiJ | 1392 | 0 |

This table indicates which strains contain a duplication of the regions on chromosome 17 from 30,174,390 – 30,651,226 Mb (Build 36). These data were generated by testing for the presence of the duplication using PCR primers as described in the text. DNA that was obtained from The Jackson Labs is indicated by the JAX Stock Number, DNA for strains not available from JAX were provided as gifts. Measurement of the duplication is not quantitative, in some cases more than two copies may be present for certain strains.
