# Supplementary material for: A Common and Unstable Copy Number Variant Is Associated with Differences in Glo1 Expression and Anxiety-Like Behavior
Source: PLoS One. 2009 Mar 6;4(3):e4649. doi: 10.1371/journal.pone.0004649 (PMC2650792; doi:10.1371/journal.pone.0004649)
Supplement: Table S4 — Significant associations between the duplication and behaviors related to activity and emotional behaviors. Shown are all phenotypes under the JAX Phenome website defined category “behavior” that are correlated with the duplication with r≥0.5 and p≤0.05. (0.20 MB DOC) [file pone.0004649.s004.doc]

**Table S4** -- **Significant associations between the duplication and behaviors related to activity and emotional behaviors.**

| **Correlated measurement from anywhere in MPD (organized by category)** | **Sex** | **Pearson r** | **p value** | **# strains** | **Spearman r** | **Log-improved** |
| --- | --- | --- | --- | --- | --- | --- |
| [**behavior – activity and motor function**](http://phenome.jax.org/pub-cgi/phenome/preview/mpdcgi?rtn=meas/catlister&req=Cbehaviorqqqactivity and motor function) | | | | | | |
| amb_long_habit Richfield1  [long-term habituation, change in ambulatory activity over days1-3](javascript:;) | male | **–0.94** | p < 0.01 | **10** | -0.64 |  |
| horiz_long_habit Richfield1  [long-term habituation, change in horizontal activity over days1-3](javascript:;) | male | **–0.94** | p < 0.01 | **10** | -0.64 |  |
| line_doseA_pre Metten1  [line crossings pre-injection](javascript:;) | female | **0.89** | p < 0.01 | **8** | 0.86 | 0.92 |
| bal19 Crabbe1  [baseline missteps crossing 19.0 mm balance beam, saline](javascript:;) | female | **–0.82** | p = 0.012 | **8** | -0.49 |  |
| line_doseB_pre Metten1  [line crossings pre-injection](javascript:;) | male | **0.79** | p = 0.019 | **8** | 0.86 |  |
| line_doseB_pre Metten1  [line crossings pre-injection](javascript:;) | female | **0.77** | p = 0.025 | **8** | 0.76 | 0.82 |
| activity_LD Brown1  [total activity (line crosses plus rearing)](javascript:;) | male | **0.76** | p < 0.01 | **13** | 0.73 |  |
| vert_long_habit Richfield1  [long-term habituation, change in vertical activity over days1-3](javascript:;) | male | **–0.75** | p = 0.013 | **10** | -0.52 |  |
| activity_LD Brown1  [total activity (line crosses plus rearing)](javascript:;) | female | **0.74** | p < 0.01 | **13** | 0.77 |  |
| activity_d1_OFT Brown1  [total locomotor activity (line crosses plus rearing), day1](javascript:;) | male | **0.73** | p < 0.01 | **13** | 0.77 |  |
| activity_d1_OFT Brown1  [total locomotor activity (line crosses plus rearing), day1](javascript:;) | female | **0.7** | p < 0.01 | **13** | 0.77 |  |
| ra_light Seburn1  [rearing, light 12h period (vertical beam breaks per minute)](javascript:;) | female | **0.67** | p < 0.01 | **14** | 0.84 | 0.77 |
| aa_light Seburn1  [ambulatory, light 12h period (beam breaks per minute)](javascript:;) | female | **0.66** | p = 0.010 | **14** | 0.75 | 0.7 |
| act_saloff Mogil2  [amount of activity with saline injection, dark 4h period (number of beam breaks)](javascript:;) | male | **0.66** | p = 0.038 | **10** | 0.7 |  |
| ra_light Seburn1  [rearing, light 12h period (vertical beam breaks per minute)](javascript:;) | male | **0.65** | p < 0.01 | **15** | 0.7 |  |
| tot_light Seburn1  [total, light 12h period (beam breaks per minute)](javascript:;) | female | **0.63** | p = 0.015 | **14** | 0.72 |  |
| dist_OFT_min2 Wahlsten1  [distance traveled, open field minute 2](javascript:;) | female | **0.59** | p < 0.01 | **21** | 0.63 |  |
| aa_light Seburn1  [ambulatory, light 12h period (beam breaks per minute)](javascript:;) | male | **0.58** | p = 0.025 | **15** | 0.65 |  |
| ra_daily Seburn1  [rearing, daily (vertical beam breaks per minute)](javascript:;) | female | **0.58** | p = 0.030 | **14** | 0.68 |  |
| speed_ave_OFT Wahlsten1  [average speed over 5 min trial](javascript:;) | female | **0.57** | p < 0.01 | **21** | 0.63 |  |
| tot_light Seburn1  [total, light 12h period (beam breaks per minute)](javascript:;) | male | **0.56** | p = 0.031 | **15** | 0.62 |  |
| dist_OFT_min2 Wahlsten1  [distance traveled, open field minute 2](javascript:;) | male | **0.55** | p < 0.01 | **21** | 0.6 | 0.61 |
| dist_OFT_min3 Wahlsten1  [distance traveled, open field minute 3](javascript:;) | female | **0.54** | p = 0.012 | **21** | 0.61 |  |
| dist_OFT_min3 Wahlsten1  [distance traveled, open field minute 3](javascript:;) | male | **0.54** | p = 0.012 | **21** | 0.62 | 0.57 |
| dist_OFT_min5 Wahlsten1  [distance traveled, open field minute 5](javascript:;) | female | **0.54** | p = 0.012 | **21** | 0.55 |  |
| speed_ave_OFT Wahlsten1  [average speed over 5 min trial](javascript:;) | male | **0.53** | p = 0.013 | **21** | 0.6 | 0.58 |
| dist_OFT_min4 Wahlsten1  [distance traveled, open field minute 4](javascript:;) | female | **0.52** | p = 0.016 | **21** | 0.57 |  |
| dist_OFT_min1 Wahlsten1  [distance traveled, open field minute 1](javascript:;) | male | **0.51** | p = 0.018 | **21** | 0.56 |  |
| [**behavior – anxiety**](http://phenome.jax.org/pub-cgi/phenome/preview/mpdcgi?rtn=meas/catlister&req=Cbehaviorqqqanxiety) | | | | | | |
| beam_breaks Flaherty1  [number beam breaks in closed quadrants](javascript:;) | male | **0.83** | p = 0.011 | **8** | 0.79 |  |
| defecation_LD Brown1  [fecal boli count](javascript:;) | female | **–0.83** | p < 0.01 | **13** | -0.59 |  |
| stretch_LD Brown1  [total streach attends](javascript:;) | male | **–0.80** | p < 0.01 | **13** | -0.55 |  |
| boli Flaherty1  [total number fecal boli](javascript:;) | male | **–0.78** | p = 0.023 | **8** | -0.36 |  |
| pct_close3 Flaherty1  [percent of time in closed quadrant 3](javascript:;) | female | **0.77** | p = 0.025 | **8** | 0.79 | 0.8 |
| closed3 Flaherty1  [time in closed quadrant 3](javascript:;) | female | **0.77** | p = 0.025 | **8** | 0.79 | 0.8 |
| pct_close3 Flaherty1  [percent of time in closed quadrant 3](javascript:;) | male | **0.73** | p = 0.041 | **8** | 0.79 |  |
| closed3 Flaherty1  [time in closed quadrant 3](javascript:;) | male | **0.73** | p = 0.041 | **8** | 0.79 |  |
| defecation_EPM Brown1  [fecal boli count](javascript:;) | female | **–0.73** | p < 0.01 | **13** | -0.53 |  |
| stretch_EZM Brown1  [total stretch attends](javascript:;) | female | **–0.71** | p = 0.021 | **10** | -0.45 |  |
| stretch_LD Brown1  [total streach attends](javascript:;) | female | **–0.68** | p = 0.010 | **13** | -0.45 |  |
| stretch_EPM Brown1  [total stretch attends](javascript:;) | female | **–0.68** | p = 0.011 | **13** | -0.45 |  |
| stretch_d1_OFT Brown1  [total stretch attends, day1](javascript:;) | female | **–0.67** | p = 0.012 | **13** | -0.52 |  |
| defecation_d1_OFT Brown1  [fecal boli count, day1](javascript:;) | female | **–0.67** | p = 0.013 | **13** | -0.41 |  |
| stretch_EZM Brown1  [total stretch attends](javascript:;) | male | **–0.67** | p = 0.017 | **12** | -0.45 |  |
| defecation_EZM Brown1  [fecal boli count](javascript:;) | female | **–0.67** | p = 0.034 | **10** | -0.48 |  |
| stretch_d1_OFT Brown1  [total stretch attends, day1](javascript:;) | male | **–0.65** | p = 0.017 | **13** | -0.55 |  |
| close_ent_EPM Brown1  [closed arms entries](javascript:;) | male | **0.61** | p = 0.034 | **12** | 0.67 |  |
| close_ent_EPM Brown1  [closed arms entries](javascript:;) | female | **0.59** | p = 0.033 | **13** | 0.66 | 0.67 |
| urination_LD Brown1  [urine puddle count](javascript:;) | female | **–0.57** | p = 0.043 | **13** | -0.35 |  |
| urination_EPM Brown1  [urine puddle count](javascript:;) | female | **–0.56** | p = 0.048 | **13** | -0.45 |  |
| [**behavior – exploratory**](http://phenome.jax.org/pub-cgi/phenome/preview/mpdcgi?rtn=meas/catlister&req=Cbehaviorqqqexploratory) | | | | | | |
| LMS Golani1  [lingering episodes, total distance between episodes per duration of episodes](javascript:;) | male | **0.74** | p = 0.037 | **8** | 0.76 |  |
| trans_LD Brown1  [transitions between light and dark zones](javascript:;) | male | **0.68** | p = 0.011 | **13** | 0.77 |  |
| trans_LD Brown1  [transitions between light and dark zones](javascript:;) | female | **0.67** | p = 0.012 | **13** | 0.77 |  |
| dist_ave_OFT Wahlsten1  [average distance traveled in 1 min](javascript:;) | female | **0.57** | p < 0.01 | **21** | 0.63 |  |
| center_ent_d1_OFT Brown1  [total center square entries, day1](javascript:;) | female | **0.56** | p = 0.045 | **13** | 0.66 | 0.64 |
| dist_ave_OFT Wahlsten1  [average distance traveled in 1 min](javascript:;) | male | **0.53** | p = 0.013 | **21** | 0.61 | 0.59 |
| [**behavior – learning and memory**](http://phenome.jax.org/pub-cgi/phenome/preview/mpdcgi?rtn=meas/catlister&req=Cbehaviorqqqlearning and memory) | | | | | | |
| vis_acuit64 Brown2  [visual acuity mean latency to reach cued platform, 0.64 threshold](javascript:;) | male | **–0.76** | p < 0.01 | **13** | -0.52 |  |
| pct_vis_detec_d8 Brown2  [visual detection % correct performance, 0.17 grating threshold, day8](javascript:;) | male | **–0.70** | p < 0.01 | **13** | -0.46 |  |
| fall_ave_d1 Brown2  [mean latency to fall, day1](javascript:;) | female | **0.64** | p = 0.034 | **11** | 0.68 |  |
| pct_pat_disc_d8 Brown2  [pattern discrimination % correct performance, 0.17 grating threshold, day8](javascript:;) | male | **–0.60** | p = 0.031 | **13** | -0.38 |  |
| rev_lat_d1 Brown2  [reversal training mean latency to escape, day1](javascript:;) | female | **–0.58** | p = 0.046 | **12** | -0.42 |  |
| [**behavior – stress reactivity**](http://phenome.jax.org/pub-cgi/phenome/preview/mpdcgi?rtn=meas/catlister&req=Cbehaviorqqqstress reactivity) | | | | | | |
| OFTactivity Gershenfeld1  [distance traveled on the open field (OFT), 1st trial (dur=5min), baseline](javascript:;) | male | **0.89** | p < 0.01 | **8** | 0.86 |  |
| OFTrearing Gershenfeld1  [vertical movements (rearing) on the open field (OFT) (dur=15min), baseline](javascript:;) | male | **0.86** | p < 0.01 | **8** | 0.86 |  |
| OFTcenter Gershenfeld1  [average center time in the open field (OFT) (2 trials each dur=5min), baseline](javascript:;) | male | **0.84** | p = 0.010 | **8** | 0.86 |  |
| LDtransits_Rx Gershenfeld1  [number of light-dark (LDB) transitions (dur=10min), after Rx (imipramine )](javascript:;) | male | **0.8** | p = 0.018 | **8** | 0.86 |  |
| OFTactivity_Rx Gershenfeld1  [distance traveled on the open field (OFT), 1st trial (dur=5min), after Rx (imipramine )](javascript:;) | male | **0.76** | p = 0.030 | **8** | 0.86 |  |
| LDtransits Gershenfeld1  [number of light-dark (LDB) transitions (dur=10min), baseline](javascript:;) | male | **0.76** | p = 0.029 | **8** | 0.76 |  |
| OFTrearing_Rx Gershenfeld1  [vertical movements (rearing) on the open field (OFT) (dur=15min), after Rx (imipramine )](javascript:;) | male | **0.74** | p = 0.034 | **8** | 0.86 |  |
| OFTcenter_Rx Gershenfeld1  [average center time in the open field (OFT) (2 trials each dur=5min), after Rx (imipramine )](javascript:;) | male | **0.73** | p = 0.038 | **8** | 0.76 |  |

Shown are all phenotypes under the JAX Phenome website defined category "behavior" that are correlated with the duplication with r≥0.5 and p≤0.05.
