# Supplementary figures and images for: A Common and Unstable Copy Number Variant Is Associated with Differences in Glo1 Expression and Anxiety-Like Behavior
Source: PLoS One. 2009 Mar 6;4(3):e4649. doi: 10.1371/journal.pone.0004649 (PMC2650792; doi:10.1371/journal.pone.0004649)

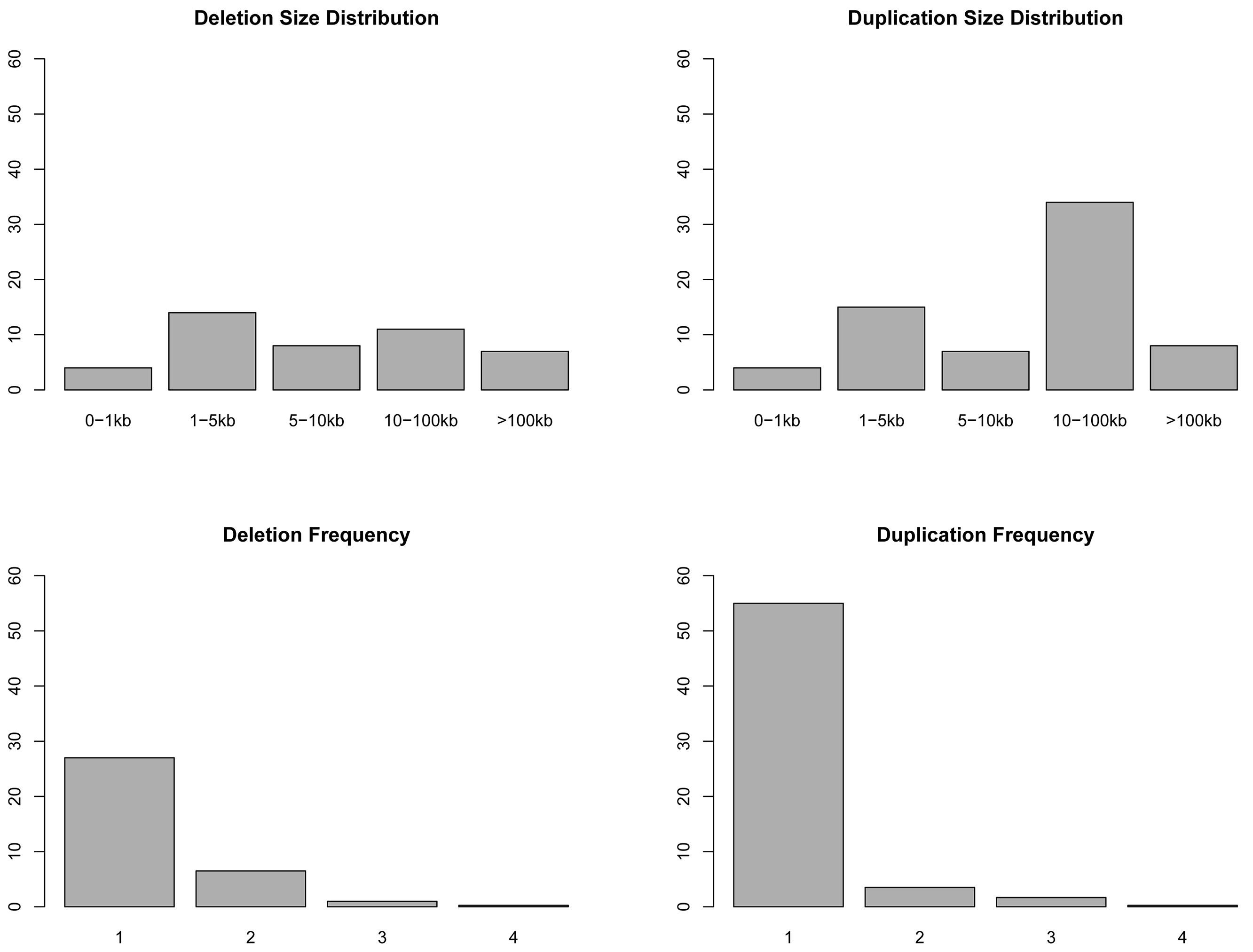

Supplement: Figure S1 — Size and frequency of duplications detected in genome-wide scan. Histograms showing frequency distribution of deletions (upper left) and duplications (upper right) as a function of size and the number of strains in which deletions (lower left) and duplications (lower left) were observed. (5.42 MB TIF) [file pone.0004649.s005.tif]
